# Effects of segmentation granularity on nutrient prediction from real-world food images

**DOI:** 10.3389/fnut.2026.1927530

**Published:** 2026-10-07

**Authors:** Liam Hand, Guido Camps, Ricardo da Silva Torres, Albert Ali Salah

**Affiliations:** 1 Division of Human Nutrition and Health, Wageningen University & Research, Wageningen, Netherlands; 2 Artificial Intelligence Group, Wageningen University & Research, Wageningen, Netherlands; 3 Information and Computer Sciences, Utrecht University, Utrecht, Netherlands

**Keywords:** automated dietary assessment, deep learning, food image analysis, food segmentation, ingredient segmentation, nutrient estimation, Nutrition5K

## Abstract

Diet quality is central to preventing non-communicable diseases, yet traditional dietary assessment methods are burdensome and error-prone. Estimating nutrients directly from food images offers a scalable alternative, but most methods treat a meal as a single visual entity and estimate calories and mass more accurately than individual macronutrients. It has not yet been systematically evaluated whether decomposing a meal into its ingredients - predicting nutrients per ingredient and aggregating to the dish level - improves estimation. We compared three input representations for nutrient regression from overhead food images: direct regression from the original image, regression from binary food-region masks, and regression from fine-grained ingredient-level masks. To enable this, we annotated a 1,056-image subset of the Nutrition5K dataset with 2,922 ingredient-level segmentation masks across 40 categories, and used an identical regression model for all conditions. We also evaluated a realistic setting with automatically generated masks. Contrary to our hypothesis, finer segmentation did not improve overall performance: the unsegmented and binary food-region segmentation conditions performed comparably, while ingredient-level segmentation resulted in the highest error. Ingredient-level predictions preserved the relative balance of macronutrients well per ingredient, but errors in each ingredient's estimated nutrient quantities accumulated once aggregated to the dish level, leading to a general, though not strict, tendency toward worse performance as dish complexity increased. Ingredient-level regression stayed competitive for simpler dishes with visually separable ingredients, but struggled when ingredients were highly mixed. These findings indicate that the principal limitation of ingredient-level nutrient estimation is accurate quantity estimation rather than modeling of nutrient composition. Future work should prioritize explicit quantity cues, such as depth or volume, to complement ingredient-level decomposition. We release our annotations and code to support reproducible research.

## 1 Introduction

Non-communicable diseases (NCDs) acount for millions of deaths worldwide each year, with total global mortality projected to reach 75.5 million by 2050 (1). Despite the multifactorial etiology of these conditions, diet quality and nutritional interventions play a critical role in their prevention and risk reduction (2). However, traditional dietary recording and assessment methods are prone to many limitations including recall bias, systematic underreporting, measurement error, and participant burden (3–6). This includes the gold standard dietary recording method—weighted food diary (7, 8)—which involves identifying each ingredient in a meal, measuring its weight or volume, and referencing nutritional databases such as the United States Department of Agriculture (USDA) food database (9) to determine nutrient composition—a burdensome, time-consuming, and impractical activity for many.

In order to address these limitations and improve the accuracy and scalability of dietary monitoring, researchers have explored automated approaches for estimating nutritional intake. However food image analysis is itself complicated by many factors. Unlike many everyday objects, food items frequently appear mixed, overlapping, or partially occluded, making precise detection and segmentation difficult (10). In addition, food ingredients exhibit high intra-class variability, as the same ingredient may appear in substantially different forms depending on preparation methods and cooking processes (11). At the same time, high inter-class similarity between visually similar foods (e.g., mashed potatoes and hummus) can further complicate classification and localization tasks (11). Food image analysis is further complicated by challenging training conditions, particularly due to the limitations and variability present in existing datasets. Food datasets typically exhibit long-tailed category distributions, where a small number of classes are heavily represented while many others contain limited samples, leading to biased model training and reduced performance on rare food types (11). In addition, background clutter, such as dishware, utensils, and table settings, can introduce noise that interferes with feature extraction (10). Variations in illumination and imaging conditions may also affect model robustness (12). Finally, the diversity of global cuisines poses a significant generalization challenge, as models trained on limited datasets often struggle to perform reliably on unseen dishes and ingredients (13). Therefore, accurately estimating nutritional content from food images remains a challenging problem.

Existing approaches to nutritional estimation from food images also largely treat the meal as a single visual entity. Zhao et al. (14) however demonstrated that incorporating a segmentation step to isolate food regions from the background improves nutrient regression performance. However, their method relies on binary food segmentation and does not explicitly model the compositional structure of meals. In practice, most dishes consist of multiple ingredients with distinct nutritional properties, suggesting that representing a meal as a single region may limit the ability of regression models to capture ingredient-specific nutrient signals.

To our knowledge, the impact of ingredient-level segmentation on nutrient regression performance has not been systematically evaluated. Recent evidence suggests that explicitly reasoning about ingredients may benefit nutritional estimation. For example, Khlaisamniang et al. (15) showed that prompting multimodal large language models to first identify ingredients before predicting nutrition improves estimation accuracy. In addition, prior work has observed that whole-image regression models tend to accumulate larger errors in macronutrient prediction compared to calorie and mass estimation (16–22). This limitation may arise because macronutrient composition depends not only on total food quantity, but also on the specific ingredients present and their relative proportions, which are not explicitly represented in global image features. Explicitly modeling ingredient structure may therefore provide a more informative representation for nutrient estimation, while also aligning more closely with traditional dietary assessment methods such as weighted food diaries. Furthermore, ingredient-level modeling may improve interpretability by enabling analysis of how individual components contribute to prediction errors. These observations motivate investigating whether explicitly modeling ingredient-level structure can improve automated nutrient estimation from food images.

To investigate this question, this study evaluates how segmentation granularity influences nutrient regression performance. Specifically, we compare three input representations for nutrient prediction: (1) direct regression from RGB images, (2) regression from binary food-region segmentation masks following the approach of Zhao et al. (14), and (3) regression from ingredient-level segmentation masks that explicitly model the compositional structure of meals. To enable this evaluation, we annotate a subset of 1,056 Nutrition5K (22) images with 2,922 ingredient-level segmentation masks covering visible ingredients across 40 categories. Nutrient values are then predicted for each segmented ingredient and aggregated to obtain dish-level estimates. Following on from the work of Zhao et al. (14) and Khlaisamniang et al. (15), we hypothesize that segmenting the food image into its visible constituent ingredients for nutrient prediction will lead to better results than direct regression and binary food-region segmentation before regression from RGB food images.

This work makes the following contributions:

1. We present the first controlled study on the Nutrition5K dataset that systematically evaluates how segmentation granularity affects nutrient regression accuracy and error behavior.
2. We establish a set of baseline comparisons that isolate the impact of segmentation granularity on nutrient estimation, extending a recent state-of-the-art (SOTA) food-region segmentation paradigm (14) to ingredient-level representations.
3. We introduce a new ingredient-level segmentation subset of the Nutrition5K dataset, consisting of 1,056 annotated images with 2,922 visible ingredient masks, enabling reproducible research on ingredient-aware nutritional estimation. We make our annotations publicly available.
4. We evaluate a practical deployment scenario by replacing ground-truth masks with predicted ingredient masks generated by a SOTA food segmentation architecture (23), enabling assessment of the proposed framework under realistic segmentation conditions.
5. We publicly release the dataset, code, and protocol to ensure reproducibility.

## 2 Related work

Several food datasets have been produced to support research in specific components of automated dietary assessment. Food-101 (24) was an early food dataset concerned with the classification of single-label food items. UNIMIB2016 (25) and UECFoodPixComplete (26) are widely used food datasets with dish-level segmentation masks. FoodSeg103 (11) provides ingredient-level segmentation masks for real-world dishes, enabling the evaluation of food segmentation models designed to handle complex ingredient-level localization tasks. Food2K (27) offers over one million images across more than one thousand food categories, supporting large-scale food recognition research. In contrast, the Nutrition5K dataset (22) focuses on nutritional estimation and contains approximately 5,000 cafeteria meals composed of over 250 ingredients, with detailed annotations including ingredient names, weights, and macronutrient values derived from the USDA database (9), as well as approximately 3,500 RGB-D images for a subset of these meals. These datasets have become widely used benchmarks for evaluating food localization and nutrient estimation systems.

Over the last decade, increasingly sophisticated deep learning (DL) models have been put forward which utilize these datasets in an attempt to solve various nutritional assessment tasks. Myers et al. (28) extended Food-101 to explore food recognition and portion estimation. In regard to food localization, Wu et al. (11) contributed their Recipe Learning Module (ReLeM) as an initial benchmark on the FoodSeg103 (29) dataset, which leveraged multimodal information to address the large visual variability of food ingredients. More recent architectures have adapted general-purpose segmentation frameworks, such as Mask2Former, to improve performance on FoodSeg103. More recently, methods incorporating foundation models, such as the Segment Anything Model (SAM), have been proposed to improve segmentation robustness and generalization to previously unseen food types (23, 30). In addition, open-vocabulary segmentation approaches have begun exploring the use of vision language models to enable recognition of novel food categories beyond the fixed label sets used during training (31–33).

Regarding overall nutrient prediction from food images, a number of studies have proposed benchmark models using the Nutrition5K (22) dataset. Thames et al. (22) provided an early baseline by training an InceptionV2 (34) network to predict total mass, calories, and macronutrients from the RGB images extracted from side-view videos. Subsequent studies explored alternative regression formulations and stronger backbone architectures to improve prediction accuracy. For example, Wang et al. (35) proposed a coarse-to-fine regression framework that first predicts discrete nutrient ranges before refining estimates through offset regression, achieving improved performance on overhead RGB images. More recent architectures combining convolutional and transformer-based feature extraction have further reduced prediction error on the benchmark (17).

In contrast to direct regression approaches, Zhao et al. (14) introduced a pipeline that incorporates an intermediate segmentation step prior to nutrient estimation. Their method first applies a UNet (36)-based model to segment food regions from the background in overhead images, before passing the resulting masked images to a ResNet-101 (37) regression network for nutrient prediction. By removing background information and focusing the regression model on food pixels, their approach achieved a benchmark score across 3,224 overhead Nutrition5K images, demonstrating that incorporating food localization can improve nutritional estimation performance compared to direct RGB regression.

Beyond RGB-only approaches, several studies have explored incorporating the depth information available in Nutrition5K to improve nutrient estimation. RGB-D fusion methods combine visual appearance with geometric cues related to food volume and portion size. For example, Shao et al. (21) proposed a multimodal feature fusion architecture that integrates RGB and depth representations for nutrient prediction, while subsequent work has explored transformer-based architectures and multi-scale feature fusion networks for more effective cross-modal learning (18, 20, 38). These multimodal approaches have generally reported improved performance compared to RGB-only models.

In addition to visual modalities, some studies have explored incorporating ingredient-level information to guide nutritional estimation. Feng et al. (39) proposed an ingredient-guided fusion network that leverages ingredient metadata available in the Nutrition5K dataset to improve feature learning for nutrient prediction. More recently, multimodal frameworks integrating RGB images, depth maps, and ingredient descriptions through vision–language learning have achieved further improvements in benchmark performance (19).

Existing approaches to nutrient prediction predominantly rely on whole-image representations, without explicitly modeling the compositional structure of meals. Consequently, the impact of decomposing food images into constituent ingredients and performing nutrient regression at the ingredient level remains largely underexplored.

## 3 Materials and methods

### 3.1 Dataset and image selection

This study utilized the overhead RGB images available in the Nutrtion5K (22) dataset. Alongside the imagery, detailed nutritional metadata, both at the dish level (mass, calories, protein, carbohydrates, and fat) and the ingredient level is provided. Ingredient masses were recorded in real time during preparation using a digital kitchen scale with ±1 g precision, and nutritional composition was computed from these masses using USDA (9) reference data. Caloric values are reported in kilocalories (kcal), and mass and macronutrients are reported in grams (g).

Of the 3,490 overhead RGB images available, a subset of 1,056 images were chosen for segmentation at the ingredient-level. The subset was obtained by simple random sampling without stratification, performed by an external annotation service. Any samples from the original Nutrition5K data containing poor lighting, framing issues, or non-food content were excluded, leaving 1,056 high-quality overhead food images for this study.

To characterize the chosen subset, an ingredient frequency analysis was conducted across the 1,056 dishes. The analysis revealed 145 unique ingredient labels, corresponding to 2,922 ingredient instances. As shown in Figure 1, the distribution followed a long-tailed pattern, with a few ingredients (e.g., carrot and broccoli) occurring frequently, while many (e.g., tuna, pizza) appeared rarely or only once. Specifically, 100 of the 145 classes contained fewer than 20 instances, and 23 classes contained only a single instance. This imbalance presents challenges for model training and evaluation, particularly for generalization to underrepresented classes.

**Figure 1:**
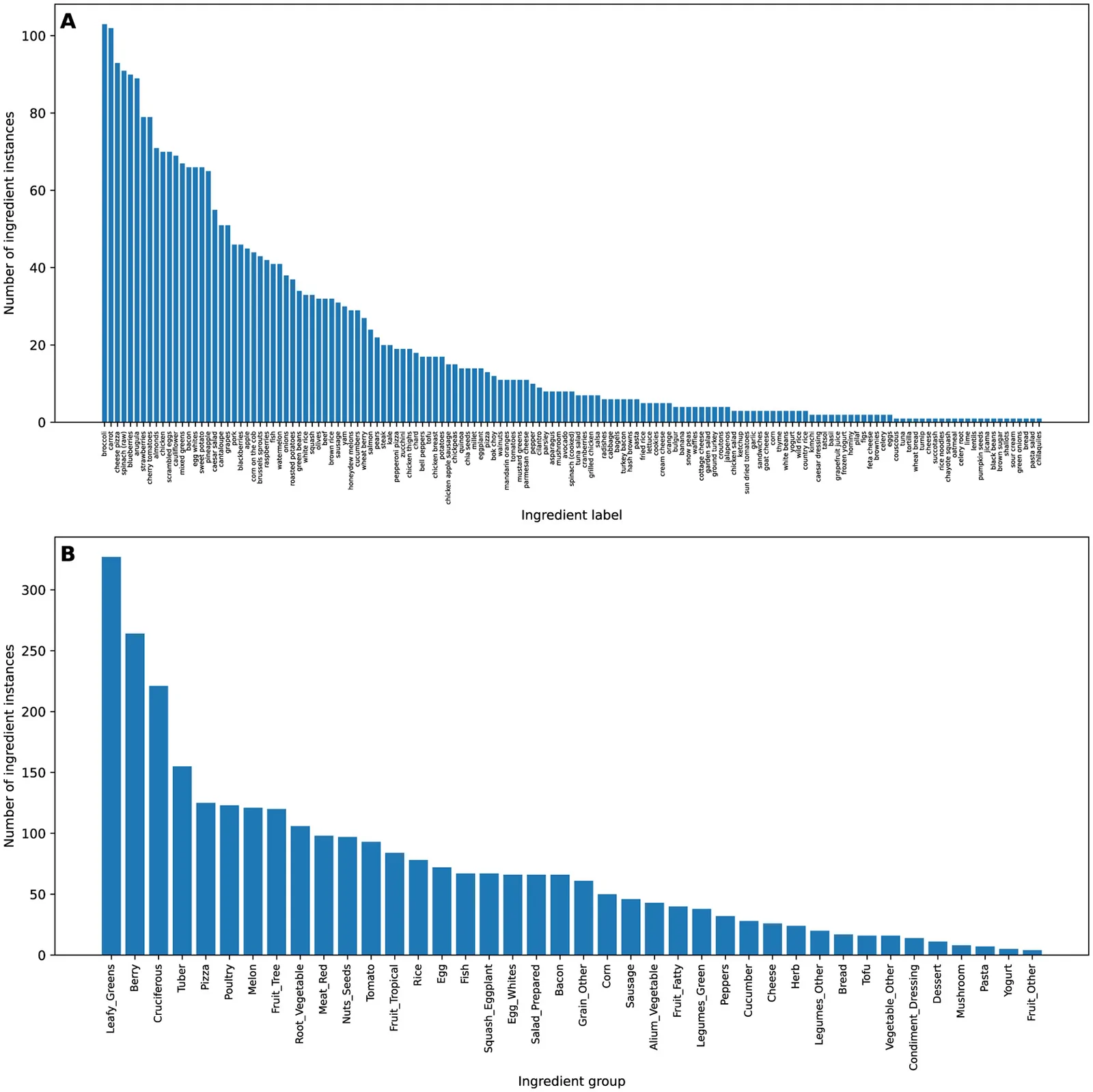
Ingredient frequency analysis across the selected 1,056 dishes. **(A)** Original distribution across 145 ingredient labels. **(B)** Distribution after consolidation into 40 nutritionally grouped categories.

To mitigate this issue, ingredient labels were consolidated into coarser groups based on nutritional similarity and semantic consistency. Groupings were guided by USDA reference values: ingredients were merged if their nutritional composition per 100 g fell within approximately 50 kcal, 10 g carbohydrates, 10 g protein, or 10 g fat of each other, and if they could be sensibly combined into a common category. For example, strawberries, blackberries, raspberries, and blueberries were grouped under *berry*; cheese pizza, pepperoni pizza, and generic pizza under *pizza*; and salmon, tuna, and other fish under *fish*. The full mapping of fine-grained to coarse labels is provided in the Supplementary Table S1. Following grouping, a second frequency analysis identified 40 unique ingredient groups. Of these, only 9 contained fewer than 20 instances, and none contained a single instance (Figure 1).

### 3.2 Ingredient segmentation annotations

Segmentation masks were generated for each visually identifiable ingredient in the 1,056 selected images. Annotations were performed on the V7 Darwin platform by professional annotators with experience in high-quality pixel-level labeling. Resolution was preserved, and multiple annotators contributed to the process. Mask labels were assigned by first checking the dish ingredient label in the Nutrition5K (22) metadata, and then reassigned to the appropriate coarse category as defined in Supplementary Table S1. Ingredients that were not visually distinguishable in RGB images (e.g., oils and sugar) were excluded from annotation. All segmentation masks underwent a final round of manual quality control to confirm boundary alignment and correct assignment to the appropriate coarse category. Example annotated dishes, where ingredient segmentation masks are overlaid on the original RGB image, are shown in Figure 2. These illustrate the visual variability of the dataset and the increasing segmentation difficulty as dish complexity grows.

**Figure 2:**
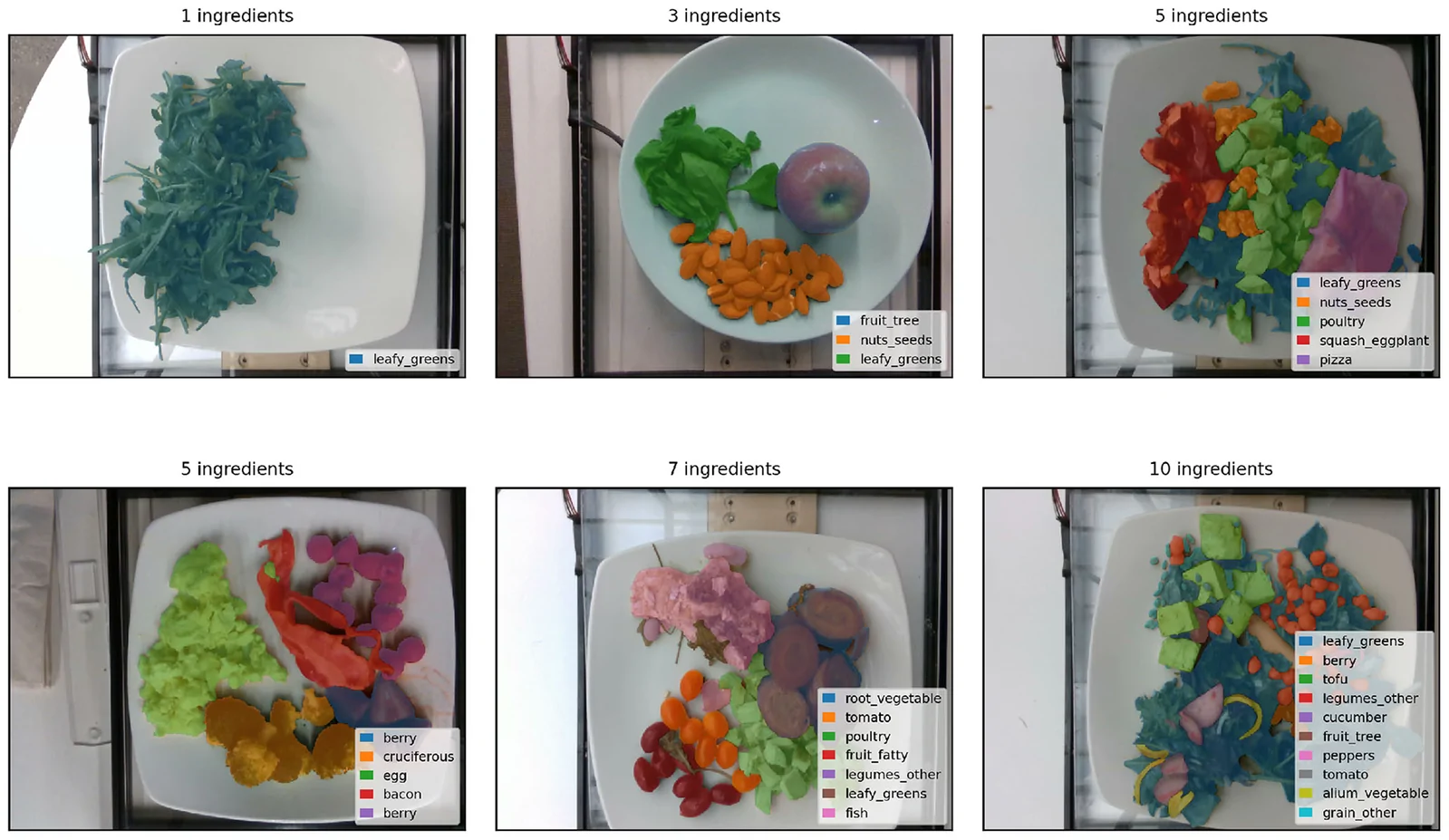
Representative examples from our ingredient-annotated Nutrition5K subset. Each panel shows the original overhead RGB image with the ingredient-level segmentation masks overlaid as semi-transparent colored regions; the legend in each panel lists the corresponding coarse ingredient categories.

### 3.3 Nutrient label structure

#### 3.3.1 Dish-level labels

In the dish-level setting (unsegmented and binary segmentation conditions), each input image corresponds to a complete meal. The ground-truth label for each sample is a five-dimensional continuous vector:


$${\text{y}}_{i}=\left[y_{i,\text{mass}},\;y_{i,\text{calories}},\;y_{i,\text{fat}},\;y_{i,\text{carb}},\;y_{i,\text{protein}}\right]\in {\mathbb{R}}^{5},$$

where mass is measured in grams (g), calories in kilocalories (kcal), and fat, carbohydrates, and protein in grams (g). These values represent the total nutritional content of the entire dish depicted in the image.

In this setting, the regression model learns a direct mapping from the full image to the aggregated nutritional quantities of the meal.

#### 3.3.2 Ingredient-level labels

In the ingredient-level setting, each sample corresponds to a single segmented ingredient instance extracted from a dish. Each ingredient instance is associated with its own five-dimensional nutrient vector:


$${\text{y}}_{k}=\left[y_{k,\text{mass}},\;y_{k,\text{calories}},\;y_{k,\text{fat}},\;y_{k,\text{carb}},\;y_{k,\text{protein}}\right]\in {\mathbb{R}}^{5},$$

where the values represent the nutritional contribution of that specific ingredient only.

Multiple ingredient instances may originate from the same dish. During ingredient-level training, each instance is treated as an independent regression sample. For dish-level evaluation under ingredient segmentation, predicted nutrient quantities are aggregated by summing predictions across all ingredient instances belonging to the same dish.

### 3.4 Architecture

For nutrient prediction, the regression architecture proposed by Zhao et al. (14) was adopted. The model consists of an ImageNet (40) pretrained ResNet-101 backbone followed by a Squeeze-and-Excitation (SE) module (41) which performs channel-wise feature recalibration, adaptively reweighting the backbone's feature channels before regression. The pooled 2,048-dimensional feature vector is then mapped to the five nutrient outputs (mass, calories, fat, carbohydrate, and protein) by three fully connected layers (2,048 → 1,024 → 256 → 5) with ReLU activations and dropout applied to the intermediate layers.

Zhao et al. (14) reported strong performance of this architecture compared to alternative convolutional and Vision Transformer backbones for overhead RGB-based nutrient prediction on the Nutrition5K dataset. To ensure a controlled comparison and isolate the effect of segmentation granularity, the same regression backbone was used across all experiments in this study.

Specifically, the model was evaluated under three input conditions: (i) unsegmented RGB images, (ii) binary food-region segmentation masks as implemented by Zhao et al. (14), and (iii) fine-grained ingredient-level segmentation proposed in this work. The model architecture is shown in Figure 3, and the three input representations are shown in Figure 4. By maintaining a consistent regression architecture, any differences in performance can be attributed to the segmentation strategy rather than changes in the prediction network. Essentially, no architectural modifications were introduced beyond adapting the input pipeline to accommodate the three segmentation conditions.

**Figure 3:**
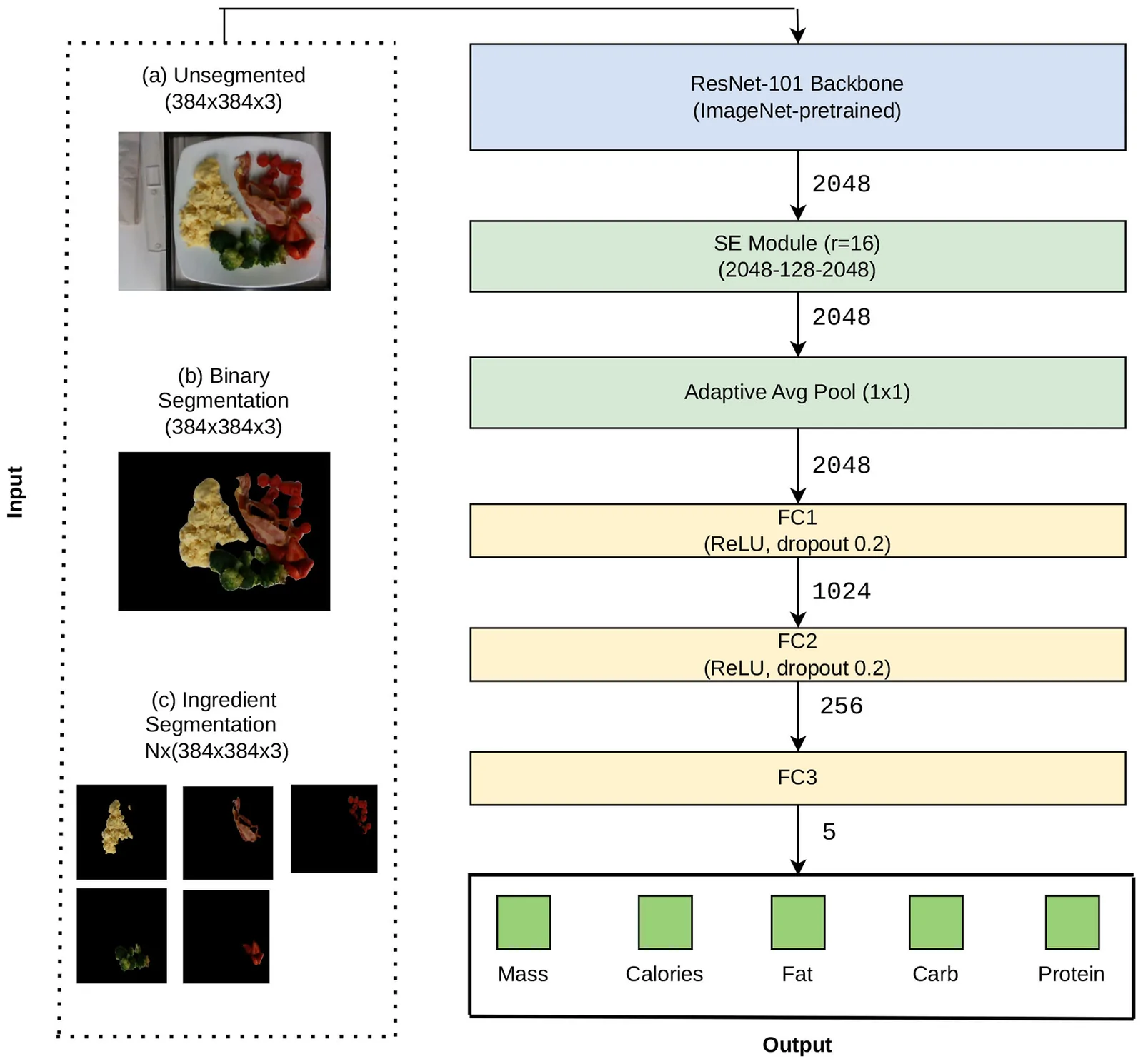
Overview of the regression architecture used across three input configurations. Unsegmented images, binary segmented images, and ingredient-level segments are independently processed by a shared ResNet-101 backbone with SE attention, followed by three fully connected layers predicting five nutritional attributes. Unsegmented and binary segmented conditions are processed as single images to obtain dish-level nutrition, while each ingredient segment is processed independently, and predictions are then summed to obtain dish-level nutrition.

**Figure 4:**
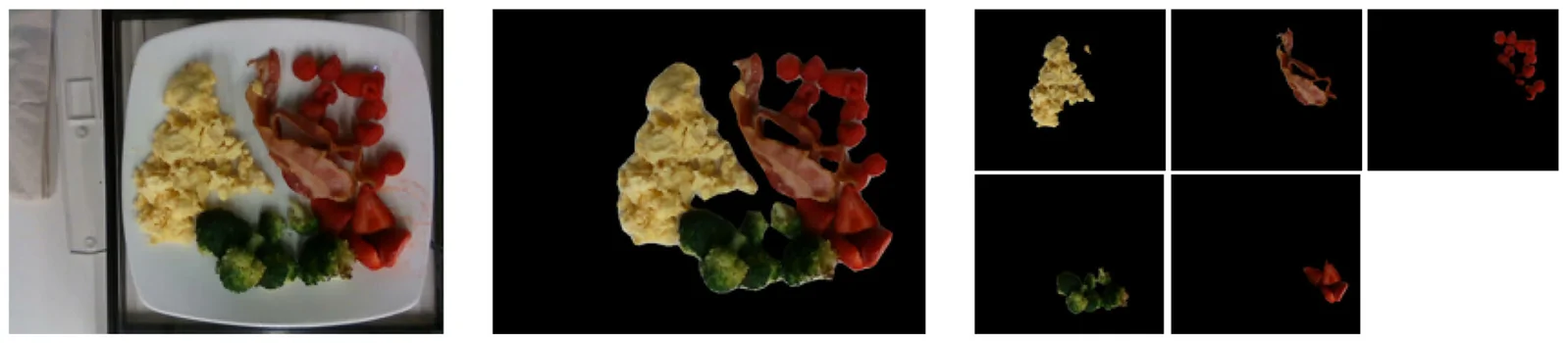
Example representations of dish_1557937157 from Nutrition5K. From left to right: (1) original RGB image, (2) binary food-region segmentation, and (3) ingredient-level segmentation showing individual ingredients (egg, bacon, berries, and cruciferous vegetables).

### 3.5 Implementation details

All models were implemented in Python 3.7 using PyTorch version 1.13, consistent with the software environment reported by Zhao et al. (14), to ensure methodological comparability. Training and inference were conducted on an AWS EC2 instance equipped with a single NVIDIA Tesla T4 GPU.

Optimization was performed using the Adam optimizer with an initial learning rate of 1 × 10^−4^, decayed exponentially by a factor of 0.99 per epoch. Default Adam parameters were retained, and no weight decay was applied. Models were trained for 300 epochs using a batch size of 8. All inputs were processed at a resolution of 384 × 384 pixels.

The complete architectural, optimisation, and preprocessing configuration, including all values not stated above, is given in Supplementary Table S2, and the full implementation is available in the accompanying repository.

### 3.6 Train-evaluation split

The dataset was partitioned at the image level using an 85:15 train–evaluation split, resulting in 898 training images and 158 evaluation images. This corresponded to 2,253 ingredient masks in the training set and 669 masks in the evaluation set, yielding an approximate mask-level distribution of 77:23.

To preserve class representation, a custom Python script was developed to approximate stratified sampling based on ingredient category frequency. Although the target ratio was 85:15, the final per-class distribution ranged between 70%–80% in the training set and 20%–30% in the evaluation set, depending on ingredient prevalence.

To ensure sufficient representation for evaluation, ingredients with fewer than 10 total instances were assigned exclusively to the training set. This threshold was selected to avoid extremely small evaluation counts that would undermine reliable statistical analysis.

For ingredient-level experiments, dish-disjoint evaluation was enforced by assigning all ingredient instances from the same dish to a single split. This prevented cross-split data leakage and ensured that performance differences could be attributed to segmentation granularity and preprocessing strategies rather than shared visual content. No images were shared between splits.

Ingredient mask frequency distributions for the training and evaluation sets are presented in Supplementary Figure S1.

### 3.7 Model selection and evaluation protocol

All experiments used the same regression architecture, optimization settings, augmentation strategy, and maximum training duration of 300 epochs. No hyperparameter tuning was performed separately for different segmentation conditions; the only experimental variable was the input representation (unsegmented, binary-segmented, or ingredient-segmented, with the corresponding preprocessing variants).

For each run, model checkpoints were monitored over training, and the checkpoint with the lowest loss on the held-out evaluation split was retained for reporting. Thus, the evaluation split was used for checkpoint selection, but not for iterative hyperparameter optimization or architecture search. Under this protocol, the reported results should be interpreted primarily as a controlled comparison of segmentation conditions under a fixed training procedure, rather than as a final estimate of out-of-sample generalization on an untouched test set.

Because all segmentation conditions were trained and selected under the same protocol and evaluated on the same held-out split, performance differences between conditions remain directly comparable.

### 3.8 Statistical analysis

As all segmentation conditions were evaluated on the same dishes, between-condition differences were assessed using paired comparisons. For each reported error metric, a 95% confidence interval was estimated by resampling dishes with replacement (10,000 bootstrap iterations) and recomputing the metric on each resample. Differences between conditions were tested using both a paired bootstrap on the difference and the Wilcoxon signed-rank test applied to per-dish errors, with statistical significance assessed at *p* < 0.05.

For dish-level complexity analysis, 95% confidence intervals for the proportion of dishes in each ingredient-count bin were computed using the Wilson score interval, and trends across ordered ingredient-count bins were assessed using the point-biserial correlation.

### 3.9 Training pipeline and nutrient prediction

Three segmentation granularities were evaluated prior to nutrient regression: (i) unsegmented RGB images, (ii) binary food-region segmentation, and (iii) fine-grained ingredient-level segmentation. The unsegmented condition served as a baseline representing direct regression from RGB food images.

For the binary segmentation condition, non-food background regions were removed using a UNet-based architecture implemented by Zhao et al. (14), who then made these cutouts publicly available. These foreground-only images were used directly as regression inputs.

For the ingredient-level condition, ground-truth masks were manually annotated for each distinguishable ingredient within an image. Each masked region was treated as an independent regression sample during training and evaluation. Metadata files preserved dish-level associations, enabling aggregation of ingredient predictions into dish-level estimates.

The regression model was trained to predict five continuous nutritional quantities: mass (g), calories (kcal), fat (g), carbohydrates (g), and protein (g). Let ŷ_*i, m*_ denote the predicted value for nutrient *m* of sample *i*, and *y*_*i, m*_ the corresponding ground-truth value.

Training optimization used a normalized mean absolute error (nMAE) loss computed per nutrient over each mini-batch. For nutrient *m*, the loss was defined as:


$$\begin{array}{l} L_{m}=\frac{\sum_{i=1}^{B}|{ŷ}_{i,m}-y_{i,m}|}{\sum_{i=1}^{B}y_{i,m}+\epsilon } \end{array}$$ (1)

where *B* denotes the batch size and ϵ is a small constant to prevent division by zero. The total loss was obtained by summing across all five nutrients:


$$\begin{array}{l} L=\overset{5}{\underset{m=1}{\sum }}L_{m}. \end{array}$$ (2)

This formulation normalizes absolute error by the total ground-truth magnitude within each batch, mitigating scale imbalance between nutrients.

#### 3.9.1 Evaluation metrics

At the dish level, predictions were evaluated using Percentage Mean Absolute Error (PMAE) as the main reporting metric. For nutrient *m*, PMAE over the evaluation set was defined as:


$$\begin{array}{l} {\text{PMAE}}_{m}=\frac{\sum_{i=1}^{N}|{ŷ}_{i,m}-y_{i,m}|}{\sum_{i=1}^{N}y_{i,m}+\epsilon }, \end{array}$$ (3)

where *N* denotes the number of evaluation samples and ϵ is a small constant to prevent division by zero. PMAE, therefore, represents the total absolute prediction error relative to the total ground-truth magnitude of the nutrient and is reported as a percentage. The mean PMAE across all nutrients was additionally reported as a summary indicator.

For ingredient-level experiments, each ingredient instance was treated as an independent regression sample and evaluated using the same metric. To obtain dish-level predictions from ingredient-level regression, nutrient estimates were summed across all ingredients belonging to the same dish:


$$\begin{array}{l} {Ŷ}_{d,m}=\overset{K_{d}}{\underset{k=1}{\sum }}{ŷ}_{k,m}, \end{array}$$ (4)

where *K*_*d*_ denotes the number of ingredient instances in dish *d*. Dish-level PMAE was then computed as:


$$\begin{array}{l} {\text{PMAE}}_{m}^{\text{dish}}=\frac{\sum_{d=1}^{D}|{Ŷ}_{d,m}-Y_{d,m}|}{\sum_{d=1}^{D}Y_{d,m}+\epsilon }, \end{array}$$ (5)

where *D* is the number of evaluated dishes and ϵ is a small constant to prevent division by zero.

#### 3.9.2 Data preprocessing

During training, data augmentation included random horizontal flips and random resized crops, followed by ImageNet normalization. During evaluation, images were resized and center-cropped prior to ImageNet normalization.

#### 3.9.3 Additional strategies for segmentation-based regression

Preliminary analysis revealed that segmented inputs contained a substantial proportion of background pixels. In the binary-segmented condition, black background regions comprised a mean of 82.1% of image pixels across the dataset (range: 52.5%–98.8%). For ingredient-level segmentation, the proportion increased to a mean of 94.5% (range: 67.8%–99.9%).

Since the regression backbone was pretrained on ImageNet (40), where background pixel distributions are diverse and non-uniform, the large homogeneous black regions in segmented images introduced a distribution shift relative to pretraining data. Furthermore, after ImageNet normalization, zero-valued background pixels are transformed into large negative activations, potentially biasing feature extraction. To mitigate this effect, two additional preprocessing strategies were evaluated. These constitute an ablation study of the input- processing pipeline: each preprocessing configuration isolates the contribution of a single component to regression performance, holding the architecture and training procedure fixed.

##### 3.9.3.1 ImageNet mean background replacement

Background pixels were replaced with the ImageNet mean RGB values prior to normalization. This approach preserved spatial structure while reducing the distributional discrepancy between segmented inputs and the pretrained backbone. Example visualizations before and after mean background replacement are shown in Supplementary Figure S2.

##### 3.9.3.2 Foreground bounding box cropping

Foreground bounding boxes were computed from segmentation masks and expanded by 1% in both spatial dimensions to prevent truncation due to mask noise. The smallest padded rectangle enclosing the dish (binary condition) or individual ingredient (ingredient-level condition) was extracted. All pixels outside this bounding box were removed, and the cropped region was resized to the original input resolution prior to regression. This strategy reduced the proportion of background pixels and constrained learning to foreground content. Example results of bounding box preprocessing are shown in Supplementary Figure S3.

##### 3.9.3.3 Combined strategy

An additional experimental condition combined mean background replacement with bounding box cropping to evaluate their joint effect on regression performance.

### 3.10 Macronutrient composition profile

In addition to evaluating nutrient prediction metrics, additional analyses were conducted to determine whether the models preserved relative macronutrient composition. These analyses were motivated by the distinction between predicting nutrient quantity and predicting nutrient structure. For example, an ingredient such as chicken may be expected to have a macronutrient profile dominated by protein, with smaller contributions from fat and carbohydrates. A model may therefore predict an incorrect absolute quantity while still preserving the relative macronutrient composition of the ingredient.

To assess this, true and predicted fat, carbohydrate, and protein values were converted into relative macronutrient profiles. For each sample, the ground-truth macronutrient ratios were calculated as:


$$\begin{array}{l} r_{fat}^{true}=\frac{y_{fat}}{y_{fat}+y_{carb}+y_{protein}}, \end{array}$$ (6)

$$\begin{array}{l} r_{carb}^{true}=\frac{y_{carb}}{y_{fat}+y_{carb}+y_{protein}}, \end{array}$$ (7)

$$\begin{array}{l} r_{protein}^{true}=\frac{y_{protein}}{y_{fat}+y_{carb}+y_{protein}}. \end{array}$$ (8)

The same calculation was applied to the predicted values:


$$\begin{array}{l} r_{fat}^{pred}=\frac{{ŷ}_{fat}}{{ŷ}_{fat}+{ŷ}_{carb}+{ŷ}_{protein}}, \end{array}$$ (9)

$$\begin{array}{l} r_{carb}^{pred}=\frac{{ŷ}_{carb}}{{ŷ}_{fat}+{ŷ}_{carb}+{ŷ}_{protein}}, \end{array}$$ (10)

$$\begin{array}{l} r_{protein}^{pred}=\frac{{ŷ}_{protein}}{{ŷ}_{fat}+{ŷ}_{carb}+{ŷ}_{protein}}. \end{array}$$ (11)

The predicted and ground-truth macronutrient profiles were then compared using macro-ratio MAE, defined as:


$$\begin{array}{l} \;\text{Macro-ratio MAE}=\\ \frac{\left|r_{fat}^{pred}-r_{fat}^{true}|+|r_{carb}^{pred}-r_{carb}^{true}|+|r_{protein}^{pred}-r_{protein}^{true}\right|}{3}. \end{array}$$ (12)

This metric represents the average proportional error across fat, carbohydrates, and protein. Unlike standard MAE or PMAE, it evaluates whether the relative macronutrient composition was preserved independently of the absolute nutrient magnitude.

Cosine similarity was also used to compare the predicted and ground-truth macronutrient profiles. For each sample, the true and predicted profiles were treated as three-dimensional vectors:


$$\begin{array}{l} {\text{r}}^{true}=\left[r_{fat}^{true},r_{carb}^{true},r_{protein}^{true}\right], \end{array}$$ (13)

$$\begin{array}{l} {\text{r}}^{pred}=\left[r_{fat}^{pred},r_{carb}^{pred},r_{protein}^{pred}\right]. \end{array}$$ (14)

Cosine similarity was then calculated as:


$$\begin{array}{l} \text{Cosine Similarity}=\frac{{\text{r}}^{true}\cdot {\text{r}}^{pred}}{||{\text{r}}^{true}||||{\text{r}}^{pred}||}. \end{array}$$ (15)

This metric evaluates whether the predicted and ground-truth macronutrient profiles point in a similar direction. Values close to 1 indicate that the predicted profile has a similar relative composition to the ground truth. Therefore, high cosine similarity indicates that the model preserved the relative balance between fat, carbohydrates, and protein, even if the total predicted nutrient quantity was inaccurate.

### 3.11 Evaluation on FoodSAM generated masks

To evaluate the effectiveness of ingredient-level segmentation in a real-world setting, ingredient masks generated by the FoodSAM model (23) were used. This experiment was designed to assess the impact of replacing ground-truth segmentation with automatically generated masks prior to nutrient regression, thereby providing a realistic baseline scenario.

FoodSAM was selected due to its strong performance on the FoodSeg103 dataset (11), achieving a mean Intersection over Union (mIoU) of 46.42%, as well as the public availability of code. The model was trained for ingredient-level segmentation using the same dataset split described in Section 3.6, following the training protocol outlined in the original work (23). This involves training a semantic segmentation transformer (SETR) (42) to predict pixel-wise ingredient labels.

During inference, each evaluation image is processed by both SAM, which generates a set of class-agnostic region proposals, and the trained SETR model, which produces a semantic segmentation map over the same image. For each SAM-generated region, the corresponding area in the SETR output is identified, and the most frequent semantic label within that region is assigned as the ingredient class. A confidence score is computed based on the overlap between the SAM region and the SETR prediction, and regions below a predefined threshold are discarded. This results in a filtered set of ingredient-level segmentation masks, where SAM provides candidate regions and SETR supplies semantic labels.

The resulting FoodSAM-generated ingredient masks were then used as input to the regression model trained on ground-truth ingredient segments under the standard setting. No additional fine-tuning of the regression model was performed. This setup replaces ground-truth segmentation with predicted masks while keeping the regression component fixed, enabling a controlled evaluation of ingredient-level segmentation under realistic conditions.

## 4 Results

### 4.1 Effect of segmentation granularity on mean PMAE

Table 1 summarizes the mean PMAE across all five nutrients under different segmentation and preprocessing conditions. The preprocessing conditions are (i) Standard (original input), (ii) IN Mean BG (replacement of background pixels with the ImageNet mean RGB values prior to normalization), (iii) BBox Only (foreground bounding-box cropping followed by resizing), and (iv) IN Mean BG + BBox (combination of ImageNet mean background replacement and bounding-box cropping). As model checkpoint selection for each condition was performed on the same evaluation split used for reporting (Section 3.7), the PMAE and MAE values reported here and in the following sections should be read as optimistic estimates of performance on unseen data rather than as unbiased benchmark figures. This does not affect the primary focus of this study, which was the controlled comparison between conditions, as the same protocol was applied identically to all conditions.

**Table 1:** PMAE (%) under different preprocessing and segmentation conditions, reported as the mean across all five nutrients and per nutrient (mass, calories, fat, carbohydrate, and protein).

Nutrient	Segmentation type	Standard	IN mean BG	BBox only	IN Mean BG + BBox
Mean	Unsegmented	19.91	-	-	-
	Binary segmentation	21.00	**20.18**	21.11	21.19
	Ingredient segmentation	**23.76**	23.92	25.40	25.62
Mass	Unsegmented	**14.73**	-	-	-
	Binary segmentation	12.46	12.44	14.14	14.84
	Ingredient segmentation	**15.24**	16.39	16.08	16.87
Calories	Unsegmented	16.73	-	-	-
	Binary segmentation	18.93	**16.93**	18.31	18.08
	Ingredient segmentation	**20.10**	21.22	23.94	24.60
Fat	Unsegmented	25.44	-	-	-
	Binary segmentation	29.08	**27.12**	27.91	27.62
	Ingredient segmentation	**33.83**	35.91	40.94	38.86
Carbohydrate	Unsegmented	**19.86**	-	-	-
	Binary segmentation	19.78	19.95	21.80	22.38
	Ingredient segmentation	22.76	**22.45**	22.59	23.17
Protein	Unsegmented	19.91	-	-	-
	Binary segmentation	24.78	24.46	23.41	**23.04**
	Ingredient segmentation	26.86	23.62	**23.43**	24.62

For each nutrient block, the best result within each segmentation type is in bold and the best overall result is in bold and underlined. Preprocessing conditions: (i) **Standard**: original input; (ii) **IN Mean BG**: replacement of background pixels with the ImageNet mean RGB values prior to normalization; (iii) **BBox Only**: foreground bounding-box cropping followed by resizing; (iv) **IN Mean BG + BBox**: combination of ImageNet mean background replacement and bounding-box cropping. A dash (-) indicates a condition that does not apply to the unsegmented baseline.

Contrary to the initial hypothesis, increasing segmentation granularity did not improve overall regression performance. The unsegmented baseline achieved the lowest mean PMAE (19.91%). Binary segmentation produced comparable but slightly higher errors across preprocessing variants, with the best binary condition (ImageNet mean background replacement) achieving 20.18%. Ingredient-level segmentation consistently resulted in substantially higher PMAE values, ranging from 23.76% (standard preprocessing) to 25.62% (ImageNet mean background replacement combined with bounding box cropping).

Paired statistical testing confirmed this ordering. Ingredient-level segmentation had significantly higher mean PMAE than the unsegmented baseline (paired difference 95% CI [1.51, 5.98], *p* = 0.001; Wilcoxon *p* = 0.012) and than binary segmentation (95% CI [0.68, 4.92], *p* = 0.010; Wilcoxon *p* = 0.043). The difference between the unsegmented and binary conditions was not statistically significant (95% CI [–0.20, 2.12], *p* = 0.102; Wilcoxon *p* = 0.96), indicating that these two conditions perform comparably while both significantly outperform ingredient-level segmentation.

To confirm that these differences were not an artifact of the single train-evaluation split, an additional five-fold cross-validation of the three conditions under standard preprocessing was performed; the ranking was preserved in every fold, with the between-condition differences remaining large relative to fold-to-fold variation (Supplementary Section 1.6, Supplementary Table S3).

Across all preprocessing conditions, the relative performance ordering remained unchanged, with unsegmented regression performing best, followed by binary segmentation and then ingredient-level segmentation. While preprocessing interventions produced only modest variation within the binary segmentation condition, ingredient-level segmentation exhibited greater sensitivity to preprocessing, particularly when bounding box foreground isolation was applied. The largest performance degradation was observed when ingredient-level segmentation was combined with bounding box cropping, resulting in an increase of approximately 5.7% relative to the unsegmented baseline.

Overall, the results indicate that finer segmentation granularity did not translate into improved nutrient regression performance under the evaluated experimental conditions.

### 4.2 Nutrient-specific performance

To further examine these trends, nutrient-level PMAE results are also reported in Table 1.

#### 4.2.1 Mass

Binary segmentation achieved the lowest mass PMAE (12.44%) (Table 1), outperforming the unsegmented baseline (14.73%). In contrast, ingredient-level segmentation consistently produced higher mass errors, with PMAE values ranging from 15.24% to 16.87%.

Within the binary segmentation condition, bounding box foreground isolation increased mass prediction error relative to both standard preprocessing and ImageNet mean background replacement. A similar trend was observed for ingredient-level segmentation, where preprocessing interventions generally led to progressive degradation in performance. The largest increase in error occurred when ingredient-level segmentation was combined with ImageNet mean background replacement and bounding box cropping.

Ingredient-level error analysis(see Supplementary Figure S4) further showed that mass prediction error was concentrated within the most common ingredients within the evaluation set—berries, cruciferous vegetables, and leafy greens—which contributed the most to total mass error.

Overall, binary segmentation provided the strongest performance for mass estimation across preprocessing conditions.

#### 4.2.2 Calories

For calorie prediction (Table 1), the unsegmented baseline achieved the lowest PMAE (16.73%). Binary segmentation with ImageNet mean background replacement yielded comparable performance (16.93%), whereas ingredient-level segmentation consistently produced higher errors across all preprocessing conditions, ranging from 20.10% to 24.60%.

Within the binary segmentation condition, all preprocessing interventions reduced PMAE relative to standard preprocessing, with ImageNet mean background replacement producing the largest improvement. In contrast, ingredient-level segmentation showed greater sensitivity to preprocessing, with all preprocessing interventions increasing calorie PMAE relative to the standard ingredient-level condition. The largest degradation occurred when ingredient-level segmentation was combined with bounding box foreground isolation.

Ingredient-level error analysis (see Supplementary Figure S4) showed that calorie prediction error was concentrated within a small number of nutrient-dense ingredient groups, including red meat, poultry, fish, and nuts/seeds.

Overall, increasing segmentation granularity did not improve calorie estimation performance under the evaluated conditions.

#### 4.2.3 Fat

Fat prediction exhibited the highest PMAE across all nutrients (Table 1). The unsegmented baseline achieved the lowest error (25.44%), while binary segmentation produced moderately higher PMAE values across preprocessing conditions. Ingredient-level segmentation resulted in substantially larger degradation, with PMAE increasing to 40.94% under bounding box preprocessing.

Unlike mass and calorie estimation, no preprocessing intervention made the segmented conditions competitive with the unsegmented baseline for fat prediction. Although some preprocessing strategies reduced error within the binary segmentation condition, fat PMAE remained higher than in the unsegmented condition. In the ingredient-level segmentation condition, all preprocessing interventions increased fat prediction error relative to standard preprocessing.

Ingredient-level error analysis (see Supplementary Figure S4) further showed that fat prediction error was dominated by fat-rich ingredient groups such as poultry, nuts/seeds, red meat, and fish, which accounted for the largest proportions of total fat error across the evaluation set.

Overall, finer segmentation granularity did not improve fat estimation performance under the evaluated conditions, and additional preprocessing was detrimental within the ingredient-level segmentation setting.

#### 4.2.4 Carbohydrates

Carbohydrate prediction (Table 1) showed the smallest difference between the unsegmented and binary segmentation conditions. The lowest PMAE was observed for binary segmentation under standard preprocessing (19.78%), which was nearly identical to the unsegmented baseline (19.86%). In contrast, ingredient-level segmentation consistently produced higher errors, with PMAE values ranging from 22.45% to 23.17%.

Within the binary segmentation condition, additional preprocessing did not improve carbohydrate prediction and bounding box foreground isolation increased error. Ingredient-level segmentation performance showed limited variation across preprocessing conditions, indicating that the proposed preprocessing techniques had little effect on carbohydrate PMAE once ingredients were isolated.

Ingredient-level error analysis (see Supplementary Figure S4) showed that carbohydrate prediction error was concentrated within carbohydrate-rich ingredient groups, particularly tubers, grain-based foods, and berries.

Overall, binary segmentation and unsegmented regression performed similarly for carbohydrate estimation, while ingredient-level segmentation consistently resulted in higher error.

#### 4.2.5 Protein

Protein prediction (Table 1) exhibited a different pattern from the other nutrients. Although the unsegmented baseline achieved the lowest overall PMAE (19.91%), preprocessing interventions within the segmented conditions produced larger relative improvements than were observed for the other nutrients. Within the binary segmentation condition, PMAE decreased progressively as additional preprocessing steps were introduced, reaching a minimum value of 23.04% under the combined ImageNet mean background replacement and bounding box setting. Ingredient-level segmentation showed a similar trend, with PMAE decreasing substantially from 26.86% under standard preprocessing to 23.43% with bounding box foreground isolation.

Despite these improvements within the segmented conditions, no segmentation strategy outperformed the unsegmented baseline. Ingredient-level error analysis (see Supplementary Figure S4) further showed that protein prediction error was dominated by protein-rich ingredient groups, particularly poultry and red meat, which contributed the largest proportions of total protein error across the evaluation set.

Overall, protein prediction appeared more responsive to preprocessing interventions within the segmented conditions than the other nutrients, although increasing segmentation granularity still did not improve performance relative to the unsegmented condition.

### 4.3 Summary of preprocessing effects

Across nutrients, the proposed preprocessing interventions produced variable effects on regression performance. ImageNet mean background replacement generally produced modest improvements within the binary segmentation condition, whereas bounding box foreground isolation increased error for mass, calorie, and carbohydrate prediction.

In the ingredient-level segmentation condition, preprocessing interventions generally reduced performance relative to standard preprocessing. This suggests that the additional background-reduction steps did not address the main source of error in the ingredient-level setting; instead, performance was often worse after preprocessing, especially for mass, calorie, and fat prediction.

Protein prediction was the main exception, showing greater responsiveness to preprocessing than the other nutrients, particularly when bounding box foreground cropping was applied.

Overall, preprocessing adjustments did not alter the relative performance ordering between segmentation strategies for mean PMAE or for most nutrient-specific outcomes: unsegmented regression remained the strongest overall condition. The main exception was calorie prediction under binary segmentation with ImageNet mean background replacement, which reduced PMAE by approximately 2% and produced performance comparable to the unsegmented baseline.

### 4.4 Effect of excluding mass and calories from PMAE evaluation

The mean PMAE metric averages errors across mass, calories, fat, carbohydrates, and protein, therefore an additional analysis was conducted to determine whether the relative performance of the models changed when evaluation was restricted to macronutrients only. This analysis excluded mass and calories from the mean PMAE calculation and averaged PMAE across fat, carbohydrates, and protein.

As shown in Table 2, removing mass and calories did not alter the overall ranking of the segmentation conditions. The unsegmented baseline remained the best-performing approach, achieving the lowest macronutrient-only mean PMAE of 21.74%. Binary segmentation produced the next-best results, with the best binary condition achieving 23.84% under ImageNet mean background replacement. Ingredient-level segmentation remained the weakest-performing approach, with its best macronutrient-only mean PMAE reaching 27.33%.

**Table 2:** Macronutrient-only mean PMAE across fat, carbohydrate, and protein.

Segmentation type	Mean PMAE (%)
Unsegmented	**21.74**
Binary segmentation	23.84
Ingredient segmentation	27.33

Mass and calories were excluded from the mean PMAE calculation. The best result is shown in bold.

These results indicate that the poorer performance of ingredient-level segmentation was not solely attributable to mass or calorie estimation. Even when the evaluation was restricted to protein, carbohydrates, and fat, finer segmentation granularity did not improve PMAE-based macronutrient prediction.

### 4.5 MAE-based analysis of dish- and ingredient-level predictions

PMAE normalizes total absolute error by the total ground-truth quantity for each nutrient, providing a scale-adjusted error measure consistent with previous Nutrition5K-based evaluations (14, 17–19, 22, 35, 38, 39, 43). However, PMAE can be sensitive to the distribution of errors across nutrients and dishes, particularly when large errors occur for nutrients with smaller total ground-truth quantities. Therefore, MAE was also examined as a complementary metric to quantify absolute prediction error. Across the nutrients, ground-truth and predicted nutrient values were z-normalized using the per-nutrient mean and standard deviation (SD) of the training set prior to error computation, placing all five nutrients on a common scale. For dish-level metrics, dish-level statistics were used, while for the ingredient-instance metrics, ingredient-level statistics were used. Additionally, macronutrient-only MAE is also reported, which considers only the mean absolute error across the protein, carbohydrate, and fat outputs. Because protein, carbohydrate, and fat share a common unit, this measurement is reported directly in grams without normalization, giving a more interpretable, scale-preserving measure.

For the unsegmented and binary segmentation models, MAE was calculated at the dish level. When all five outputs were considered (i.e., mass, calories, protein, carbohydrate, and fat), both the unsegmented and binary segmentation models achieved an MAE of 0.25 SD units (SD across dishes = 0.20), indicating near-identical performance. When the analysis was restricted to macronutrients only, the unsegmented model achieved an MAE of 4.08 g, while the binary segmentation model achieved an MAE of 4.30 g.

For the ingredient-level model, MAE was first calculated at the level of individual ingredient instances. To achieve this, per-ingredient errors were normalized using ingredient-level training statistics and are reported on that scale. Because these per-ingredient values are normalized using ingredient-level statistics and computed over individual ingredient instances rather than whole dishes, they are not directly comparable in magnitude to the dish-level metrics—however a dish-level summed-error metric (calculated below) provides the like-for-like dish-level comparison. For each ingredient, the normalized errors across all five nutrient outputs were averaged, and the resulting values were then averaged across all ingredient samples. This produced an ingredient-level MAE of 0.18 SD units. The ingredient-level macronutrient-only MAE was 1.48 g.

To examine how ingredient-level errors accumulated at dish level, and to make the ingredient model directly comparable to the dish-level metrics, a stricter summed-error metric was also calculated. For each dish and each nutrient, the absolute errors of all constituent ingredient instances were summed in their original units, so that over- and under-predictions across ingredients could not cancel out. Each per-nutrient summed error was then divided by the corresponding dish-level training SD, and the resulting normalized values were averaged across the five nutrients to obtain the dish-level summed-error MAE. Normalizing each nutrient before averaging places the five nutrients on a common scale, while using the dish-level SD keeps this metric directly comparable to the unsegmented and binary models. Under this formulation, the ingredient-level approach produced a dish-level summed-error MAE of 0.29 SD units (SD across dishes = 0.25) when all five nutrients were considered. When the analysis was restricted to macronutrients only, an MAE of 4.89 g was calculated.

Table 3 summarizes these results. The ingredient-level model produced relatively low errors when evaluated per ingredient - reflecting in part the smaller nutrient quantities of individual ingredients - however these errors increased substantially once accumulated to dish level, where a like-for-like comparison with the other models was made possible. This suggests that although individual ingredient predictions may be reasonably accurate in absolute terms, errors can accumulate across multiple ingredient instances, limiting the effectiveness of ingredient-level regression for final dish-level nutrient estimation. Per-dish errors were highly variable, with a standard deviation across dishes of approximately 0.20 for both the unsegmented and binary models and 0.25 for the ingredient summed-error metric—comparable in magnitude to the mean errors themselves (0.25, 0.25, and 0.29, respectively). Relative to this dish-to-dish variation, the difference between the unsegmented and binary models was negligible, indicating that binary segmentation provided no benefit over the unsegmented baseline.

**Table 3:** MAE-based comparison across model conditions.

Condition	Samples	All-nutrient MAE	Macronutrient-only MAE (g)
Unsegmented	158	**0.25 (0.20)**	**4.08**
Binary segmentation	158	**0.25 (0.20)**	4.30
Ingredient summed error	158	0.29 (0.25)	4.89
*Reported on a different scale:*			
Ingredient instance	638	0.18	1.48

All-nutrient MAE was computed across mass, calories, protein, carbohydrate, and fat after z-normalizing ground-truth and predicted values by the per-nutrient training mean and SD, and is reported in SD units as mean (SD across dishes). Macronutrient-only MAE was computed across protein, carbohydrate, and fat, which share a common unit, and is reported directly in grams. The *Samples* column gives the number of whole dishes (158) for the dish-level rows and the number of individual ingredient instances (638) for the ingredient-instance row. Best values among the directly comparable dish-level conditions are shown in bold. The ingredient-instance row is normalized using ingredient-level statistics and computed over single ingredients rather than whole dishes; it is therefore not directly comparable to the dish-level rows and is reported separately.

Paired significance testing supported this interpretation: for both the z-normalized all-nutrient MAE and the macronutrient-only MAE, the ingredient summed-error condition was significantly worse than the unsegmented and binary conditions, whereas the unsegmented and binary conditions were statistically indistinguishable.

As reflected in these tests, the ingredient-level summed error was consistently ranked weakest across both MAE and PMAE, indicating that ingredient-level decomposition degraded dish-level nutrient prediction. Overall, the MAE-based analysis was consistent with the PMAE results: finer segmentation did not improve dish-level nutrient prediction, and ingredient-level decomposition consistently reduced it.

### 4.6 Dish-level error analysis

To further examine model behavior beyond aggregate evaluation metrics, a dish-level error analysis was conducted. This analysis identified dishes for which each model achieved the lowest error, as well as cases where one model substantially outperformed another. In addition, dishes were grouped according to the number of ingredient instances they contained in order to assess whether dish complexity influenced model performance. For each dish, performance was evaluated using MAE across all five nutrient outputs (mass, calories, fat, carbohydrates, and protein) and using macronutrient-only MAE across fat, carbohydrates, and protein.

This analysis was intended to determine whether segmentation-based approaches were more effective for particular types of dishes, such as simpler meals with fewer ingredients, and whether ingredient-level prediction errors increased as the number of components in a dish increased.

#### 4.6.1 Ingredient-level segmentation vs. unsegmented regression

The relative performance of ingredient-level segmentation and unsegmented regression varied with the number of ingredient instances in each dish. For complete nutrient prediction, including mass, calories, fat, carbohydrates, and protein, the ingredient-level model achieved lower dish-level MAE than the unsegmented model in 54.8% of one-ingredient dishes and 44.4% of two-ingredient dishes. For dishes containing 3–4 ingredients, the ingredient-level model outperformed the unsegmented model in 53.5% of cases. However, this proportion decreased for more complex dishes, with ingredient-level segmentation outperforming unsegmented regression in only 39.5% of dishes with 5–6 ingredients and 32.1% of dishes with 7 or more ingredients.

When the analysis was restricted to macronutrients only, the ingredient-level model achieved lower MAE than the unsegmented model in 58.1% of one-ingredient dishes and 55.6% of two-ingredient dishes. However, this advantage declined for dishes with 3–4 and 5–6 ingredients, where ingredient-level segmentation outperformed unsegmented regression in 44.2% and 28.9% of cases, respectively. For dishes with 7 or more ingredients, the ingredient-level model achieved lower macronutrient-only MAE in 39.3% of cases.

These results suggest that ingredient-level segmentation was most competitive for simpler dishes containing fewer ingredients. In contrast, its relative performance generally decreased for dishes with more ingredient instances. To assess whether these apparent declines were statistically supported rather than artifacts of small bin sizes, Wilson 95% confidence intervals for each bin proportion (Table 4) were computed, and the point-biserial correlation was applied to test for a trend across ingredient counts. The trend was statistically significant for ingredient vs. unsegmented in the macronutrient-only predictions (*r* = −0.17, *p* = 0.029), but not significant for ingredient vs. unsegmented in all nutrient predictions (*r* = −0.15, *p* = 0.058). The per-bin confidence intervals are wide and overlapping, so the declining ingredient-model performance should be read as a general tendency rather than a strict monotonic decline.

**Table 4:** Percentage of dishes in which ingredient-level segmentation outperformed the unsegmented and binary segmentation models, stratified by number of ingredient instances per dish.

Ingr. count	Dishes	vs. unseg. All	vs. unseg. macro	vs. binary All	vs. binary macro
1	31	54.8 [37.8, 70.8]	58.1 [40.8, 73.6]	54.8 [37.8, 70.8]	64.5 [46.9, 78.9]
2	18	44.4 [24.6, 66.3]	55.6 [33.7, 75.4]	55.6 [33.7, 75.4]	61.1 [38.6, 79.7]
3–4	43	53.5 [38.9, 67.5]	44.2 [30.4, 58.9]	46.5 [32.5, 61.1]	41.9 [28.4, 56.7]
5–6	38	39.5 [25.6, 55.3]	28.9 [17.0, 44.8]	39.5 [25.6, 55.3]	39.5 [25.6, 55.3]
7+	28	32.1 [17.9, 50.7]	39.3 [23.6, 57.6]	32.1 [17.9, 50.7]	32.1 [17.9, 50.7]
*Trend* *p*		0.058	0.029	0.038	0.005

Wilson 95% confidence intervals are given in brackets. “All” refers to MAE averaged across mass, calories, fat, carbohydrate, and protein on z-normalized values (Section 4.5); “Macro” refers to MAE averaged across fat, carbohydrate and protein only, reported in grams without normalization, as these share a common unit. Values represent the percentage of dishes for which the ingredient-level model achieved a lower MAE than the comparison model, with Wilson 95% confidence intervals. Trend *p* is from a point-biserial correlation of the per-dish win indicator against ingredient count.

Altogether, these results are suggestive of a pattern where ingredient-level errors accumulate as dish complexity increases, potentially reducing the effectiveness of ingredient-level regression for more complex meals.

#### 4.6.2 Ingredient-level segmentation vs. binary segmentation

A similar dish-level comparison was conducted between the ingredient-level and binary segmentation models. For complete nutrient prediction, including mass, calories, fat, carbohydrates, and protein, the ingredient-level model achieved lower MAE than the binary segmentation model in 54.8% of one-ingredient dishes and 55.6% of two-ingredient dishes. However, this proportion decreased as dish complexity increased. For dishes containing 3–4 ingredients, ingredient-level segmentation outperformed binary segmentation in 46.5% of cases, decreasing to 39.5% for dishes with 5–6 ingredients and 32.1% for dishes with 7 or more ingredients.

When the analysis was restricted to macronutrients only, ingredient-level segmentation was more competitive for simpler dishes. The ingredient-level model achieved lower macronutrient-only MAE than the binary segmentation model in 64.5% of one-ingredient dishes and 61.1% of two-ingredient dishes. However, this advantage was not maintained for dishes with more ingredients, with ingredient-level segmentation outperforming binary segmentation in 41.9% of dishes with 3–4 ingredients, 39.5% of dishes with 5–6 ingredients, and 32.1% of dishes with 7 or more ingredients.

These results suggest that ingredient-level segmentation may be more competitive than binary segmentation for simpler dishes, particularly when evaluating macronutrients only. However, its relative performance decreased as the number of ingredient instances increased, again suggesting that ingredient-level errors accumulate in more complex dishes. This trend was statistically significant for both nutrient-set evaluations (vs. all-nutrient, *r* = −0.17, *p* = 0.038 and vs. macronutrient-only, *r* = −0.22, *p* = 0.005). However, the per-bin confidence intervals are wide and overlapping, so this should be read as a general declining tendency rather than a strict monotonic decline. Table 4 summarizes these results.

### 4.7 Macronutrient composition profile analysis

Macronutrient profile composition was calculated using mean-macro ratio MAE and cosine similarity, as outlined in Section 3.10.

At the ingredient-instance level, the ingredient-level model achieved a mean macro-ratio MAE of 0.06 and a mean macro-profile cosine similarity of 0.96. These results indicate that the model preserved the relative macronutrient composition of individual ingredients to a high degree, despite the poorer absolute quantity estimation observed in the PMAE and MAE analyses.

The same profile-based metrics were also calculated at dish level for the unsegmented, binary segmentation, and ingredient-level models. For the ingredient-level model, predictions were first aggregated to dish level before calculating the macronutrient profile metrics, allowing comparison with the unsegmented and binary segmentation models. At the dish level, the unsegmented model achieved a mean macro-ratio MAE of 0.06 and a mean cosine similarity of 0.97. The binary segmentation model achieved a mean macro-ratio MAE of 0.07 and a mean cosine similarity of 0.97. In comparison, the aggregated ingredient-level model achieved a mean macro-ratio MAE of 0.08 and a mean cosine similarity of 0.95.

These results suggest that the ingredient-level model preserved macronutrient composition well at the level of individual ingredient instances. However, this profile preservation did not fully translate to the aggregated dish-level predictions, where the unsegmented and binary segmentation models achieved slightly stronger profile metrics. This indicates that although the ingredient-level model often captured the relative macronutrient structure of individual ingredients, errors in estimating the relative quantity or contribution of each ingredient may have reduced the accuracy of the final dish-level macronutrient profile. Table 5 summarizes these results.

**Table 5:** Macronutrient profile similarity across segmentation conditions.

Condition	Samples	Mean ratio MAE	Mean cosine similarity
Unsegmented	158	0.06	0.97
Binary segmentation	158	0.07	0.97
Ingredient aggregated to dish	158	0.08	0.95
Computed over individual ingredient instances (not directly comparable to dish-level rows):			
Ingredient instance	638	0.06	0.96

Ratio MAE compares predicted and ground-truth fat, carbohydrate, and protein proportions, while cosine similarity measures profile alignment. The *Samples* column gives the number of whole dishes (158) for the dish-level rows and the number of individual ingredient instances (638) for the ingredient-instance row. Ingredient Aggregated to Dish was computed after summing ingredient-level predictions to dish level.

### 4.8 Ingredient-specific error contribution

Ingredient-level error analysis showed that the prediction error was not uniformly distributed across ingredient groups. Instead, a relatively small subset of nutrient-dense ingredients contributed disproportionately to total model error (Figure 5). Poultry contributed the largest proportion of total error (9.46%), followed by red meat (7.71%) and grain-based foods (6.66%). Additional contributors included tubers, nuts and seeds, cruciferous vegetables, fish, berries, and cheese, each accounting for between 5.78% and 4.20% of total error. In contrast, highly represented but lower-nutrient-density ingredients, such as leafy greens, contributed comparatively less to overall error despite their higher frequency within the dataset.

**Figure 5:**
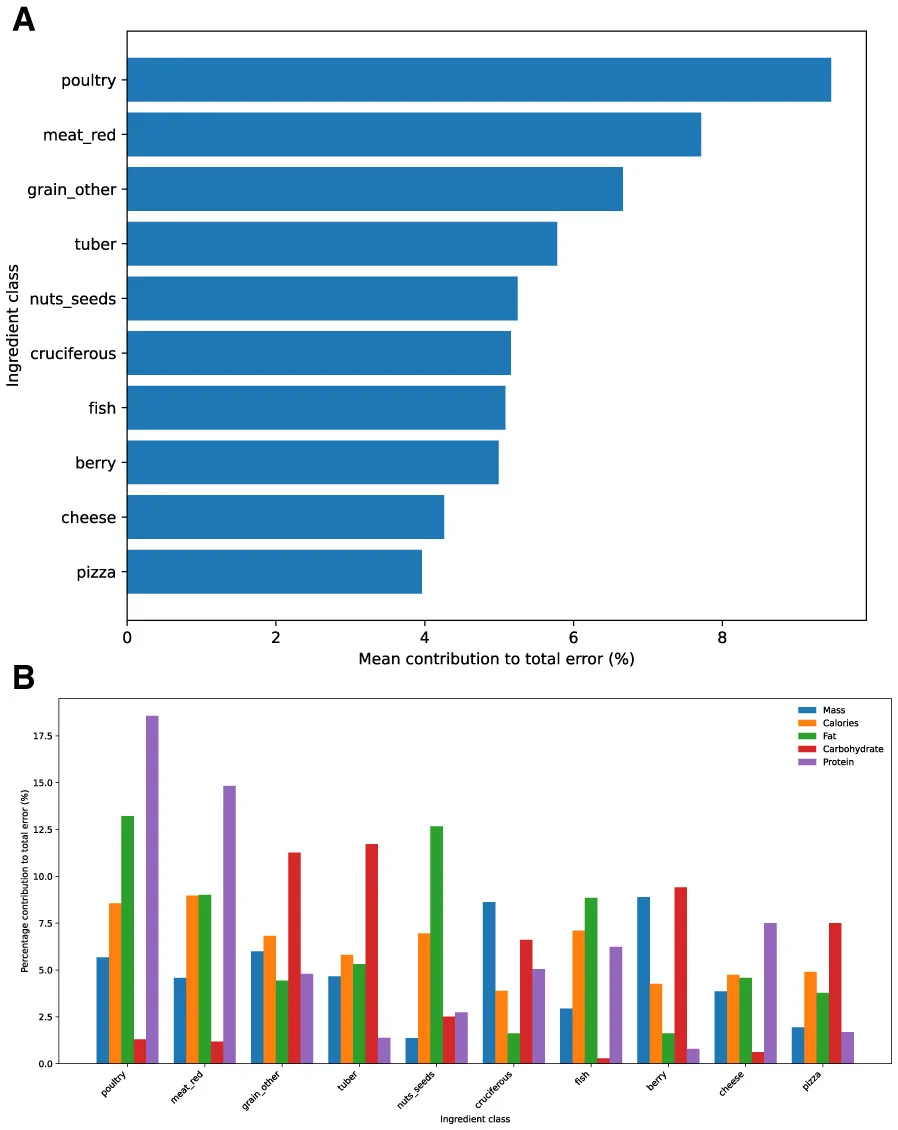
Top 10 ingredient groups contributing to model error. **(A)** Overall contribution of each ingredient group to total model error. **(B)** Contribution of the top 10 error-contributing ingredient groups decomposed by nutrient.

When decomposed by nutrient (Figure 5), ingredient groups tended to contribute most strongly to errors in compositionally related nutrients. For example, protein-rich foods contributed most strongly to protein prediction error, while carbohydrate-rich foods contributed most strongly to carbohydrate prediction error. These results indicate that prediction error is structured across ingredient–nutrient combinations rather than uniformly distributed. Combined with the macronutrient composition profile analysis (Section 4.7), which showed strong macronutrient profile similarity scores for the ingredient-level model, these findings suggest that the model captures coarse relationships between ingredient appearance and macronutrient composition despite substantial quantity estimation error.

The top 10 ingredient groups contributing to total prediction error for each nutrient are additionally shown in Supplementary Figure S4.

### 4.9 Real-world baseline

In addition to experiments using ground-truth segmentation masks, ingredient masks generated by the state-of-the-art food segmentation model FoodSAM (23) were evaluated to provide a real-world baseline for ingredient-level segmentation prior to nutrient regression, as described in Section 3.11. FoodSAM was trained and evaluated using the same dataset split outlined in Section 3.6, and dish-disjoint evaluation was maintained. Segmentation performance is reported in Supplementary Tables S4, S5.

The generated masks were used as input to the regression model trained on ground-truth ingredient masks under the standard setting. The resulting nutrient prediction performance is shown in Table 6. The mean PMAE increased to 30.17%, indicating a substantial degradation compared to the use of ground-truth masks. Similarly, performance decreased across all nutrients, with PMAE values of 27.16% for mass, 21.75% for calories, 36.74% for fat, 34.24% for carbohydrates, and 30.98% for protein.

**Table 6:** Mean and per-nutrient PMAE (%) obtained using FoodSAM-generated ingredient masks under the standard condition (no preprocessing applied prior to regression).

Mean PMAE	Mass PMAE	Calorie PMAE	Fat PMAE	Carbohydrate PMAE	Protein PMAE
30.17	27.16	21.75	36.74	34.24	30.98

Compared to results obtained using ground-truth ingredient masks, the magnitude of degradation varies across nutrients. Mass prediction exhibits the largest increase in error (15.24% to 27.16%), followed by carbohydrates (22.76% to 34.24%). In contrast, calorie prediction shows a relatively smaller change (20.10% to 21.75%), while fat and protein prediction exhibit moderate increases (33.83% to 36.74% and 26.86% to 30.98%, respectively).

These results provide a baseline estimate of performance when using automatically generated ingredient segmentation in a real-world setting, and highlight the sensitivity of ingredient-level regression performance to segmentation quality. However, as these results are specific to the masks produced by FoodSAM, the degree of degradation reflects the characteristics of this particular segmentation model; different segmentation models, or FoodSAM configured or fine-tuned differently, may yield different results.

### 4.10 Summary of main findings

Overall, increasing segmentation granularity did not improve nutrient regression performance on the evaluated subset. Across PMAE- and MAE-based evaluations, the unsegmented model consistently achieved the strongest overall dish-level performance (though its advantage over binary segmentation was not statistically significant), while ingredient-level segmentation generally resulted in higher prediction error. Although binary segmentation produced competitive results for certain nutrients, particularly mass and protein prediction, finer-grained ingredient segmentation did not improve final dish-level nutrient estimation.

Additional analyses provided further insight into the nature of these errors. Excluding mass and calories from the PMAE calculation did not alter the relative ranking of the segmentation strategies, indicating that the poorer performance of ingredient-level segmentation was not driven solely by mass or calorie prediction. Ingredient-instance MAE and macronutrient composition profile analyses further showed that the ingredient-level model often preserved coarse macronutrient structure at the individual ingredient level, achieving strong macro-profile similarity despite weaker absolute quantity estimation. However, dish-level aggregation analyses demonstrated that these errors accumulated as the number of ingredient instances increased, reducing performance for more complex meals.

Together, these findings indicate that the primary limitation of ingredient-level regression from RGB imagery lies not in modeling coarse macronutrient composition, but in accurately estimating the relative quantity and contribution of multiple ingredients within a dish. Within the current experimental setting, finer spatial decomposition of meals did not translate into improved dish-level nutrient estimation from overhead RGB images.

## 5 Discussion

### 5.1 General discussion

The primary aim of this study was to investigate the impact of segmentation granularity on nutrient regression from real-world overhead RGB food images. To this end, a ResNet-101-based regression model was trained and evaluated under three conditions: (1) unsegmented, where raw RGB images were used directly; (2) binary segmentation, where non-food background regions were removed; and (3) ingredient-level segmentation, where each image was decomposed into visible constituent ingredients, with nutrient predictions generated per ingredient and subsequently aggregated to obtain dish-level estimates. All conditions were evaluated on the same subset of the Nutrition5K (22) dataset using identical training configurations. For the ingredient-level setting, the Nutrition5K subset was manually annotated with ingredient-level segmentation masks, which were paired with the existing ground-truth ingredient mass and macronutrient metadata. These annotations were then used as input to the ingredient-level regression pipeline. Primary performance was assessed using mean percentage mean absolute error (PMAE). The unsegmented and binary segmentation conditions achieved comparable overall error, with no statistically significant difference between them, while ingredient-level segmentation yielded significantly higher error. These findings were contrary to the original hypothesis and, under our protocol, did not replicate the improvement from binary food-region segmentation as reported by Zhao et al. (14). However, additional analyses revealed that the poorer dish-level performance of ingredient-level segmentation was driven primarily by error accumulation across multiple ingredients rather than an inability to model coarse ingredient composition.

Inspired by prior Nutrition5K studies reporting substantially lower error for calorie and mass prediction than for macronutrient prediction (16–19), additional analyses were conducted to investigate whether segmentation granularity influenced the modeling of macronutrient composition independently of calories and mass. This is important for practical dietary assessment systems, as accurate macronutrient estimation can be more nutritionally informative than direct prediction of calories or food mass, primarily because caloric values can be derived directly from macronutrient quantities. Furthermore, food mass itself is primarily useful as an intermediate quantity for estimating nutrient composition.

To investigate this, supplementary analyses were performed using both all-nutrient evaluation and macronutrient-only evaluation. However, removing mass and calories from the PMAE calculation did not alter the relative ranking of the segmentation conditions, with the unsegmented and binary models performing comparably and ingredient-level segmentation remaining the weakest. Similar trends were observed in the MAE-based analyses. Although the ingredient-level model produced higher dish-level MAE overall, the difference between the unsegmented and ingredient-level models was reduced under macronutrient-only evaluation, showing that ingredient-level regression was comparatively more competitive when restricted to fat, carbohydrate, and protein prediction.

To investigate whether the ingredient-level model preserved nutritional structure, additional macronutrient composition analyses were performed using macro-ratio MAE and cosine similarity, as outlined in Section 4.7. At the ingredient-instance level, the ingredient-level model achieved composition profile metrics comparable to the unsegmented model and slightly stronger than the binary segmentation model. However, performance deteriorated when ingredient-level predictions were aggregated to dish level, where the unsegmented and binary models achieved stronger profile preservation. These findings show that the ingredient-level model is often able to preserve the relative balance between fat, carbohydrates, and protein within individual ingredient predictions, but struggles to accurately estimate the relative quantity and contribution of ingredients when aggregating predictions across a complete meal. This further indicates that the primary limitation of ingredient-level regression lies not in modeling broad nutritional structure, but in accurately estimating and aggregating quantity-related information across multiple ingredients within a dish.

Further insight into the behavior of the ingredient-level model was obtained through the dish-level complexity analysis. As shown in Table 4, the ingredient-level model achieved competitive performance for simpler meals containing fewer ingredient instances, but performance progressively deteriorated as dish complexity increased. For complete nutrient prediction, including mass and calories, the ingredient-level model outperformed the unsegmented model in a greater proportion of dishes containing four or fewer ingredients, with the exception of the two-ingredient category. When evaluation was restricted to macronutrients only, the ingredient-level model performed better than the unsegmented model for dishes containing one or two ingredients. However, this relative advantage declined substantially as additional ingredients were introduced into the dish. This overall trend was also observed when comparing ingredient-level model behavior with the binary segmented model behavior. Across both comparisons, this behavior is explained by the accumulation of small over- and under-predictions across individual ingredient instances. While many ingredient-level predictions may be reasonably accurate in isolation, aggregation of these absolute errors across multiple ingredients leads to progressively larger dish-level estimation error.

The visual presentation of ingredients within a dish also influences the effectiveness of ingredient-level regression. While the dish-level analysis demonstrated a general decline in performance as the number of ingredient instances increased, ingredient count alone does not fully explain the observed behavior. The degree to which ingredients are visually separable is also an important factor. When ingredients are spatially distinct and occupy relatively homogeneous regions, the ingredient-level model can achieve highly competitive performance and, in some cases, outperform both the unsegmented and binary segmentation models. This is illustrated in Figure 6, where a dish containing five ingredients is easily decomposed into visually distinct components, allowing the ingredient-level model to generate accurate ingredient-instance predictions. This produces competitive total-dish nutrient estimates after aggregation despite the relatively high ingredient count, illustrating that ingredient-level regression can perform well when ingredient boundaries are clearly defined and ingredient predictions can be aggregated with limited error accumulation.

**Figure 6:**
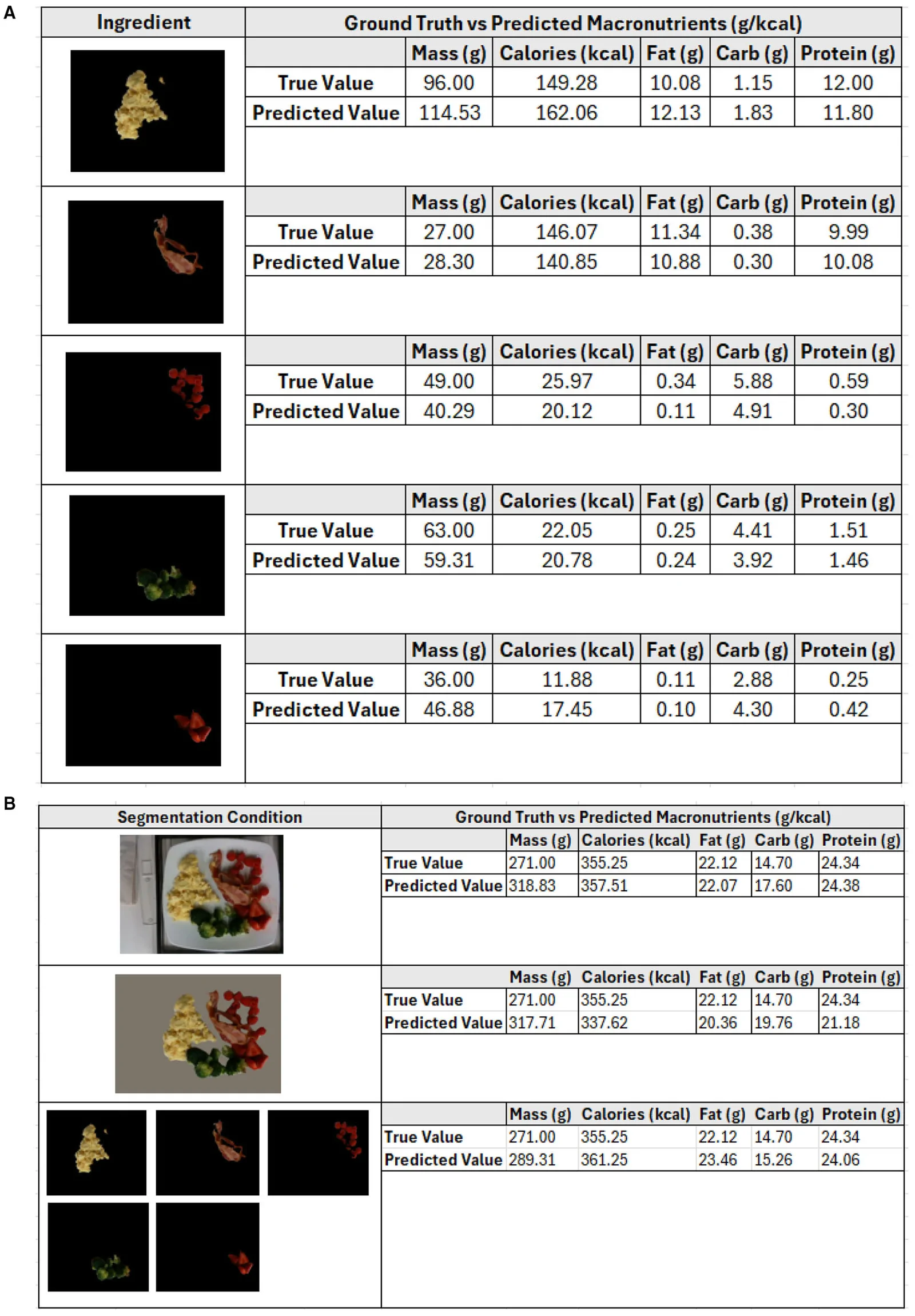
Example nutrient predictions for a visually separable dish containing five ingredients. **(A)** Predicted and ground-truth nutrient values for each segmented ingredient instance. **(B)** Predicted and ground-truth dish-level values under the unsegmented, binary segmentation, and ingredient-level segmentation conditions.

In contrast, dishes containing heavily mixed, overlapping, occluded, or non-homogeneous ingredients present a more challenging scenario for ingredient-level prediction. As shown in Figure 7, the visual boundaries between ingredients become increasingly ambiguous while the individual ingredients are also non-homogeneous, potentially reducing the accuracy of both segmentation and subsequent nutrient estimation. Under these conditions, the unsegmented and binary segmentation models benefit from access to broader contextual cues that are removed during ingredient decomposition. The ingredient-level model suffers from greater quantitative errors which are then accumulated across the dish. This example illustrates that ingredient-level regression suffers when ingredient boundaries are not clearly defined or when individual ingredients are spread out.

**Figure 7:**
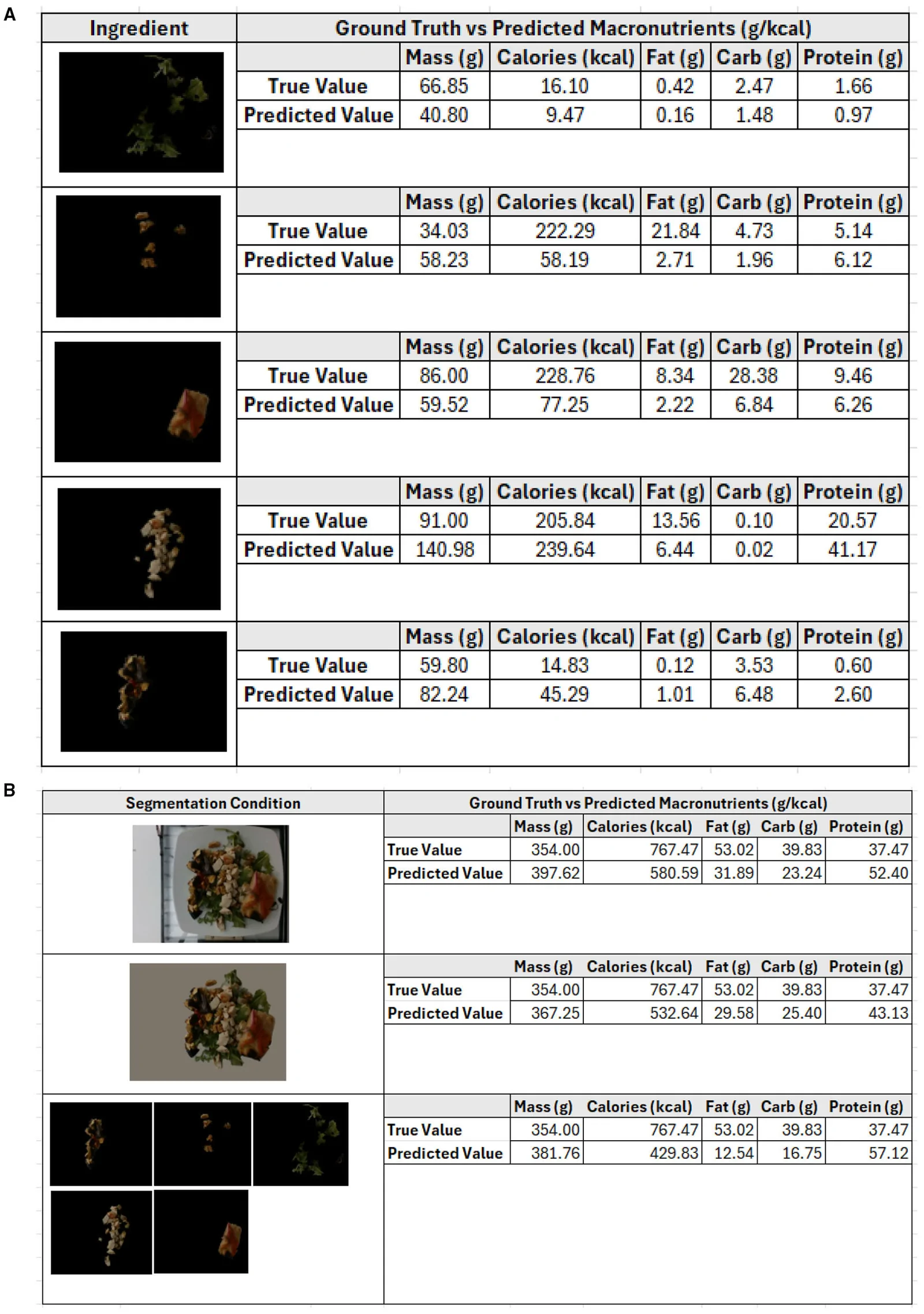
Example nutrient predictions for a dish with heavily mixed, overlapping, or non-homogeneous ingredients. **(A)** Predicted and ground-truth nutrient values for each segmented ingredient instance. **(B)** Predicted and ground-truth dish-level values under the three segmentation conditions.

Collectively, these observations show that ingredient separability, in addition to ingredient count, is an important determinant of ingredient-level regression performance and provide further evidence that the effectiveness of ingredient decomposition depends on the visual structure of the meal.

Altogether, these findings further support that the inferior dish-level performance of ingredient-level nutrient regression arises primarily from cumulative aggregation error rather than a complete inability to model coarse nutritional structure at the ingredient level. The results show that ingredient-level regression remains competitive for relatively simple meals with a small number of visible ingredients, but becomes increasingly unstable as dish complexity increases and errors accumulate across multiple ingredient predictions. Nevertheless, ingredient-level modeling retains an important advantage in interpretability and explainability, as nutrient predictions can be attributed directly to individual ingredients—a direct contrast to the case of unsegmented or binary images, where nutrients and quantity are inferred implicitly using additional signals such as plate size, food arrangement within the plate, and/or food co-occurrence in the image. Also, the ingredient-level prediction setting may amplify inherent limitations of RGB imagery. Nutritionally significant components such as oils, dressings, and absorbed ingredients are often partially or entirely invisible, making accurate estimation at the ingredient level particularly challenging. Indeed, this is suggested in Table 1, as the difference in error for fat prediction between the models is much higher in the ingredient-level setting than for any other nutrient. Also, the ingredient-level condition will be more sensitive to segmentation-mask noise, as shown by our real-world baseline using FoodSAM-generated masks; it is noted however that this baseline reflects the performance of one specific segmentation model, and results may differ with other automatic segmentation methods.

In regard to the preprocessing strategies outlined in Section 3.9.3, only the application of an ImageNet mean background fill to binary-segmented inputs resulted in improved performance. This is due to the distribution shift introduced by binary segmentation relative to the ImageNet pre-training data used for the ResNet-101 backbone. In the standard binary segmentation setting, large regions of zero-valued (black) background pixels remain. These are not representative of natural image statistics and thus adversely affect feature extraction (44), confirming that preserving compatibility with the pre-trained feature distribution is more beneficial than aggressively removing background information in binary segmentation pipelines.

In contrast, bounding box cropping generally resulted in poorer performance than retaining the original image context, particularly in the ingredient-level segmentation condition. This finding is contrary to the initial hypothesis that removing “non-informative" background pixels may improve regression performance by focusing the model on the food itself. Our findings indicate that in the uncropped setting, images preserve information about the relative size and spatial extent of ingredients, which provide an important cue for implicit quantity estimation. When cropped ingredient regions are resized to a fixed input resolution, much of this scale information is removed. This interpretation is consistent with the inductive biases of convolutional neural networks, which are sensitive to spatial structure but do not possess inherent scale invariance. Furthermore, the cropping and resizing process may introduce interpolation artifacts and amplify the effects of segmentation inaccuracies, particularly for smaller ingredient regions. These findings highlight that preserving spatial cues related to ingredient size is important for nutrient regression and that relative scale acts as an important implicit supervisory signal for quantity estimation in RGB-based models.

### 5.2 Comparison to other studies

Zhao et al. (14) reported that applying binary food-region segmentation prior to regression improved nutrient prediction performance on Nutrition5K overhead RGB images compared to direct regression from unprocessed images. Building upon this prior work, we introduced an additional level of granularity by decomposing each food image into its constituent ingredients and performing nutrient regression at the ingredient level, followed by aggregation to obtain dish-level predictions. Contrary to the initial hypothesis, our results demonstrate that increasing segmentation granularity led to a progressive degradation in performance. The degradation in ingredient-level performance is explained via the cumulative nutrient prediction error aggregation across dishes with multiple ingredients, as explored in the previous section. One potential explanation for the degradation within the binary segmentation condition was the difference in dataset size—our experiments were conducted on a subset of 1,056 images, compared to the 3,224 images used by Zhao et al. (14). However, we performed additional experiments with the full 3,224-image subset between unsegmented and binary segmented inputs, and the same trend was observed - unsegmented inputs continued to outperform segmented counterparts. with the unsegmented condition achieving a mean PMAE of 14.99% compared to 17.19% for the binary condition. This is reported in Supplementary Table S6, where it is also shown that the unsegmented condition produced lower error on all five nutrient outputs. As ingredient masks were only annotated for the 1,056-image subset, the ingredient-level condition could not be included in this replication. Overall lower error values are observed in the 3,224-image replication compared to those of the 1,056-image subset reported in Table 1, which is consistent with larger training set size. The two experiments can be therefore compared only in terms of the relative ordering of conditions rather than quantitative numbers, however these results further indicate that the reported improvement using binary segmentation observed by Zhao et al. (14) does not replicate under our protocol. This discrepancy may be attributable to implementation-specific factors, such as differences in training–test partitioning, preprocessing pipelines, and training configurations, which are not fully specified in Zhao et al. (14), and therefore cannot be ruled out as contributing factors. Alternatively, unsegmented images retain contextual cues—such as plate boundaries, food arrangement, and implicit portion size indicators that are informative for nutrient estimation, which are removed in the binary segmentation condition.

In addition to Zhao et al. (14), several other studies have evaluated nutrient prediction using the RGB modality of the Nutrition5K dataset (17, 22, 35). While results are not directly comparable due to differences in dataset splits and experimental setups, several consistent trends emerge. In particular, fat is consistently reported as the most challenging nutrient to predict, followed by carbohydrates and protein, whereas calorie and mass estimation exhibit comparatively lower error. The findings of the present study and our ingredient-level model closely align with these observations.

The present work provides additional insight into the underlying causes of these trends through explicit evaluation of segmentation granularity. If prediction performance were primarily limited by the ability to identify or isolate ingredients, finer-grained segmentation would be expected to improve results by providing more precise spatial information. Instead, our results reaffirm that the dominant challenge in nutrient prediction from RGB imagery does not lie in the ability of our models to capture coarse nutritional structure, but rather in estimating the physical quantity of each ingredient. This interpretation is consistent with the observed nutrient-specific trends. Fat prediction remains particularly difficult, as it is often influenced by non-visible components (e.g., oils) that are difficult to capture in RGB images. Similarly, although carbohydrates and protein are associated with visually identifiable ingredients, accurate estimation depends on reliable inference of portion size, which is hindered when spatial context is reduced. This confirms that future progress in vision-based dietary assessment should focus less on improving spatial decomposition and more on incorporating explicit mechanisms for quantity estimation.

Beyond RGB-only approaches, Ma et al. (38) and Kwan et al. (18) explored the incorporation of depth information available within the Nutrition5K dataset to address the limitations of quantity estimation from RGB imagery alone. Among these approaches, only Kwan et al. (18) achieved a substantial improvement over RGB-only methods in overall PMAE. Interestingly, the benefits of depth information were observed primarily in mass and calorie prediction, while improvements in individual macronutrient prediction were comparatively modest, suggesting that depth information contributes most strongly to the estimation of physical quantity rather than nutritional composition. The ingredient-level analyses from our study indicate that modeling coarse nutritional structure is often possible from RGB imagery alone, whereas accurate estimation of quantity remains the dominant source of error. Consequently, future research may benefit from combining ingredient-level decomposition with explicit quantity-estimation signals such as depth information. Such an approach could allow ingredient-level models to leverage the compositional advantages of ingredient isolation while compensating for the quantity-estimation limitations observed in RGB-only regression. More broadly, these findings again suggest that future progress in ingredient-level nutrient estimation may depend less on improving ingredient identification and more on developing mechanisms that explicitly model ingredient quantity and volume.

In addition to visual modalities, several studies have explored incorporating ingredient-level information to improve nutrient estimation. Approaches such as Feng et al. (39) and Kuang et al. (43) integrate ingredient metadata with visual features, allowing models to account for nutritionally relevant components that may be visually ambiguous or not directly observable. Similarly, Feng et al. (19) leverages ingredient text during training to guide representation learning, improving alignment between visual features and nutritional content. These multimodal approaches outperform both RGB-only and RGB-D models in terms of mean PMAE on the Nutrition5K dataset, with ingredient-informed methods showing particularly strong improvements in protein and carbohydrate prediction. These findings suggest a promising future research direction that combines ingredient-level decomposition with ingredient-guided semantic information. While the present study indicates that ingredient-level segmentation alone does not resolve the challenge of quantity estimation, the addition of ingredient-level semantic cues may improve the modeling of nutritional composition by providing explicit information about ingredient identity and expected nutrient profiles. Such an approach could enable future systems to jointly reason about ingredient appearance, composition, and quantity, potentially overcoming some of the limitations observed in RGB-only ingredient-level regression. Table 7 summarizes the results of these studies alongside the modalities employed.

**Table 7:** Comparison of nutrient prediction performance across prior studies using the Nutrition5K dataset.

References	Modalities	Mean	Mass	Calorie	Fat	Carb	Protein
Zhao et al. (14)	RGB only	17.06	11.75	15.68	21.41	18.79	17.68
Wang et al. (17)	RGB only	17.80	N/A	14.72	18.04	19.84	15.40
Ma et al. (38)	RGB-D	17.30	10.30	14.00	22.60	19.50	20.20
Kwan et al. (18)	RGB-D	15.65	8.72	12.80	19.67	18.66	18.42
Feng et al. (39)	RGB-D + Text	15.90	9.80	13.70	19.20	19.30	17.60
Kuang et al. (43)	RGB-D + Text	16.80	11.20	14.90	19.70	18.90	19.50
Feng et al. (19)	RGB-D + Text	14.40	9.90	13.80	18.90	14.60	15.00

All methods are evaluated on overhead food images, with some approaches incorporating additional modalities such as depth or text-based ingredient information. Performance is reported in terms of mean PMAE across nutrients. These methods are reported for context rather than direct comparison to the present study. All predict nutrients from whole overhead images, frequently using additional modalities (depth or text) and the full Nutrition5K dataset, whereas the present study uses an RGB-only, ingredient-segmented 1,056-image subset to isolate segmentation granularity. Absolute error values are therefore not directly comparable across studies due to differences in task formulation, modality, and dataset composition.

### 5.3 Strengths and limitations

This study makes several contributions at both the methodological and resource levels. To the best of our knowledge, it is the first work to systematically investigate ingredient-level segmentation as a precursor to nutrient regression from food images. Furthermore, we establish a baseline for this formulation under both idealized conditions—using ground-truth ingredient masks—and more realistic conditions, using automatically generated masks from a state-of-the-art segmentation model (23), enabling a comprehensive assessment of the potential and limitations of fine-grained decomposition for nutrient prediction. Finally, all code, annotations, and supporting materials are publicly released to promote reproducibility and support future research in this area. Overall, this addresses a gap in the literature, where the impact of fine-grained segmentation on nutrient regression has not previously been evaluated in a controlled setting.

However, some experimental limitations should be considered. First, all experiments were conducted using RGB imagery alone. While this design enabled a controlled evaluation of segmentation granularity, our results show that a major challenge in nutrient estimation lies in accurately inferring ingredient quantity and aggregating predictions across multiple ingredients within a dish. Although the ingredient-level formulation is consistent with the weighted food diary gold standard nutritional analysis (7, 8), where total nutritional content is obtained by summing the contributions of individual ingredients, the present findings indicate that utilizing this approach in automated systems depends critically on improving quantity estimation. Also, as dish complexity increases, small errors in individual ingredient predictions accumulate and adversely affect dish-level performance. Consequently, the full potential of ingredient-level nutrient prediction may require the incorporation of additional modalities that provide explicit quantity-related information, such as depth maps, ingredient metadata, volumetric cues, 3D reconstruction techniques, or multimodal semantic representations.

Second, a single set of hyperparameters was used across all experimental conditions to ensure a controlled and fair comparison between segmentation strategies. While this strengthens the validity of the comparative analysis, it may not reflect the optimal configuration for each individual condition. Future work could investigate condition-specific optimisation strategies, alternative loss functions, and architectures designed specifically for ingredient-level prediction and aggregation. Furthermore, the preprocessing experiments suggest that ImageNet-pre-trained backbones may be sensitive to distribution shifts introduced by segmentation-based inputs. Future research could therefore explore food-specific pre-training, large-scale food foundation models, or self-supervised representation learning approaches such as DINO (45) to improve feature extraction for food understanding tasks.

A further limitation concerns our model selection protocol. For each condition, the checkpoint with the lowest loss on the held-out evaluation split was retained, and performance was then reported on that same split. Because this evaluation set was not used for hyperparameter optimisation or architecture search, and because the same protocol was applied identically to all three conditions, this supports a controlled comparison between segmentation conditions rather than an estimate of how well any single model generalizes to unseen data. Therefore, the absolute PMAE and MAE values reported should be regarded as optimistic estimates of true performance on unseen data, and treated with caution when compared against benchmark figures obtained on untouched test sets. A separate test set was not reserved as the annotated subset was not large enough to partition into distinct training, validation, and test sets while retaining sufficient per-class representation for reliable evaluation (Section 3.6). However, 5-fold cross-validation under standard preprocessing was performed as an additional test (see Supplementary Section 1.6), and this showed that the segmentation ranking was preserved in every fold, with the between-condition differences remaining large relative to fold-to-fold variation. Therefore, while the absolute error values reported herein should be regarded as optimistic estimates, the ordering of the segmentation conditions appears robust, as it persisted across all cross-validation folds. Future work with a larger annotated dataset could employ a separate validation split for checkpoint selection and an untouched test set for unbiased performance estimation, which would additionally permit the condition-specific hyperparameter optimisation discussed above.

Furthermore, the Nutrition5K dataset comprises US cafeteria-style meals consisting largely of Western dishes, and therefore under-represents dishes and ingredients that may be popular in other culinary traditions, such as many Eastern cuisines. As our segmentation-based approach depends on ingredients being visually distinct and spatially separable, it may be poorly suited to cuisines built around inherently mixed or combined preparations, such as curries, stews, and casseroles. In such dishes, ingredients are frequently mixed, layered, or obscured by sauces, to the extent that even a human observer may struggle to identify the constituent ingredients from an overhead image alone. These cases may therefore require additional information modalities, such as ingredient text or other semantic cues, for the model to determine ingredient identity and quantity. Addressing these cases represents an important avenue for future research that the present model does not attempt to tackle, and evaluating segmentation granularity across a broader range of cuisines and plating styles would help establish the generality of our findings.

An additional consideration relates to the prediction targets themselves. In the present study, all models were trained to jointly predict mass, calories, fat, carbohydrates, and protein, consistent with previous Nutrition5K-based work. However, macronutrient-focused evaluation may provide a more direct assessment of nutritional understanding, as caloric values can be derived directly from macronutrient quantities. Future work could therefore investigate architectures trained specifically for macronutrient prediction, rather than joint prediction of all nutritional outputs, to determine whether this leads to improved modeling of nutritionally relevant information and reduces the influence of quantity-related targets such as mass on the optimisation process.

Regarding dataset contributions, this work introduces a novel dataset that augments the Nutrition5K images with 2,922 ingredient-level segmentation masks and corresponding ground-truth nutritional values. To our knowledge, this is the first dataset to jointly provide fine-grained spatial annotations and quantitative nutritional labels at this scale. This will enable the exploration of new research directions, including ingredient-level nutrient estimation and fine-grained reasoning over food composition, which have previously been limited by the lack of suitable annotated data. This dataset can also provide a foundation for future studies on advanced modeling approaches, including multimodal methods and improved quantity estimation techniques. In particular, the dataset can facilitate investigation into methods that explicitly model portion size and ingredient-level contributions to total nutritional content. However, it also represents a relatively small subset of the Nutrition5K dataset with a pronounced long-tailed distribution. To mitigate data sparsity, visually distinct ingredients were grouped into coarser categories based on nutritional similarity. While this improves statistical coverage, it increases intra-class visual variability, requiring the model to learn mappings from heterogeneous visual appearances to a shared nutritional representation.

To assess whether this grouping independently increased task difficulty, the separability of the merged sub-classes in the model's feature space was quantified using the silhouette coefficient (46) and visualized with t-SNE (47) projections of representative groups (see Supplementary Table S7, Supplementary Figure S5). Across the 34 multi-class groups, the original sub-classes were largely not separable (median silhouette −0.003), indicating that grouping did not force the model to distinguish visually distinct inputs. This is further supported by a per-sub-ingredient breakdown of the ingredient-level model's error (see Supplementary Table S8), which shows that visually distinct sub-ingredients within a merged class are predicted with similar error. The coarse grouping of sub-classes is therefore unlikely to be the cause of the worse ingredient-level result. This is also consistent with the ingredient-level analyses (Section 4.7; Figure 6) which show that the coarse groupings did not prevent the model from capturing broad macronutrient composition patterns. These results nonetheless cannot fully separate the effects of segmentation granularity and label grouping within the present design, and annotation at finer granularity remains a valuable direction for future confirmation. However, the present results indicate that, for macronutrient prediction, nutritional similarity is a more important consideration for ingredient grouping than fine-grained visual or taxonomic distinctions. Conversely, such groupings may be less appropriate for tasks involving micronutrient estimation, cooking-method recognition, or detailed ingredient identification, where finer-grained annotations may be required.

A further limitation is that annotations were restricted to visible ingredients, whereas the ground-truth nutritional values include contributions from non-visible components such as absorbed oils, sauces, and preparation-related factors. This introduces an unavoidable mismatch between the visual information available to the model and the nutritional targets it is required to predict.

Additionally, inter-annotator agreement was not formally quantified for the ingredient-level segmentation masks. Although annotations were produced by professional annotators and underwent a final round of manual quality control (Section 3.2), multiple annotators contributed to the dataset, and formal agreement metrics such as IoU or Dice between annotators were not computed.

Finally, the dataset consists solely of single-view RGB images. Future work could explore the use of the multi-view video sequences and depth information available within Nutrition5K to provide additional geometric and volumetric cues for quantity estimation. Expanding the scale and diversity of ingredient-level annotations, improving class balance, and investigating learning strategies specifically designed for long-tailed distributions may also further improve the utility of the dataset for ingredient-level nutrient prediction research.

## 6 Conclusion

This study investigated the effect of segmentation granularity on nutrient prediction from real-world RGB food images. Contrary to the initial hypothesis, increasing segmentation detail did not improve prediction performance. Across the evaluated conditions, the unsegmented approach achieved the lowest overall error, followed by binary segmentation, while ingredient-level segmentation produced the highest error. Notably, under our protocol, the previously reported benefit of binary food-region segmentation over unsegmented input was not replicated, which is attributed to unspecified differences in experimental configuration.

Despite this, the results showed that ingredient-level modeling was often able to preserve the broad macronutrient composition of individual ingredients and remained competitive for meals containing a small number of ingredients, and more complex meals containing a higher number of visually distinct ingredients. Performance deteriorated primarily as dish complexity increased, indicating that the main challenge lies not in representing ingredient composition, but in accurately estimating quantity when ingredients are nonhomogeneous, and in combining predictions across multiple ingredients. These findings further suggest that the current ingredient-level approach is most suitable for meals in which ingredients are clearly separated and visually distinct, whereas dish-level RGB-based approaches remain more effective for complex dishes containing many mixed or overlapping components.

The results confirm that the principal limitation of RGB-based nutrient prediction is quantity estimation. While ingredient-level decomposition provides a more interpretable representation of a meal and enables nutrient predictions to be attributed to individual ingredients, it also increases sensitivity to errors in quantity estimation and aggregation. Future work should therefore focus on incorporating explicit quantity-related information, such as depth or volumetric cues, and on developing methods that better account for the accumulation of errors across multiple ingredients.

Overall, this work provides the first systematic evaluation of ingredient-level segmentation before nutrient regression, establishes benchmark results for this setting using both ground-truth and automatically generated ingredient masks, and introduces a new ingredient-level extension of Nutrition5K. The findings confirm that simply increasing segmentation granularity is insufficient for improving nutrient prediction and that future progress will depend on better modeling of ingredient quantity rather than finer visual decomposition alone.

## Data Availability

The ingredient-level segmentation annotations and the code generated for this study are available at https://github.com/liamhand/ingredient-level-nutrition-regression. This work builds on the publicly available Nutrition5K dataset (22) and the binary-segmented images released by Zhao et al. (14), which are linked from the repository.

## Supplementary material

Supplementary Material Supporting tables and figures including the ingredient-label mapping, implementation configuration, cross-validation results, feature-space separability analysis, per-sub-ingredient error breakdown, and FoodSAM segmentation performance.
