# Supplementary material for: Effects of segmentation granularity on nutrient prediction from real-world food images
Source: Front Nutr. 2026 Oct 7;13:1927530. doi: 10.3389/fnut.2026.1927530 (PMC13683130; doi:10.3389/fnut.2026.1927530)
Supplement: Supplementary file 1 [file Supplementary_file_1.pdf]

Supplementary Material

1 SUPPLEMENTARY FIGURES AND TABLES

1.1 Ingredient Groupings

Supplementary Table S1 provides the complete mapping from the original 145 ingredient labels to the 40 nutritionally grouped categories used in the ingredient-level regression experiments. These groupings were determined using USDA reference nutritional values and semantic consistency criteria. The mapping is provided to ensure full reproducibility of label consolidation decisions.

Table S1: Complete mapping of 145 original ingredient labels to 40 coarser categories.

Original Label	Original Instance Count	New Label	New Instance Count
green onions	1	alium_vegetable	43
onions	38	alium_vegetable	
shallots	1	alium_vegetable	
garlic	3	alium_vegetable	
bacon	66	bacon	66
strawberries	79	berry	264
raspberries	42	berry	
cranberries	7	berry	
blueberries	90	berry	
blackberries	46	berry	
bread	1	bread	17
wheat bread	1	bread	
bagels	6	bread	
tortilla	1	bread	
sandwiches	3	bread	
chilaquiles	1	bread	
croutons	4	bread	
parmesan cheese	11	cheese	26
goat cheese	3	cheese	
feta cheese	2	cheese	
cream cheese	5	cheese	
cottage cheese	4	cheese	
cheese	1	cheese	
caesar dressing	2	condiment_dressing	14
sour cream	1	condiment_dressing	
ketchup	3	condiment_dressing	
brown sugar	1	condiment_dressing	
salsa	7	condiment_dressing	

Continued on next page

Original Label	Instance Count	New Label	Instance Count
corn	3	corn	50
corn on the cob	44	corn	
succotash	1	corn	
hominy	2	corn	
broccoli	103	cruciferous	221
cauliflower	69	cruciferous	
brussels sprouts	43	cruciferous	
cabbage	6	cruciferous	
cucumbers	29	cucumber	29
cookies	5	dessert	11
brownies	2	dessert	
waffles	4	dessert	
scrambled eggs	70	egg	72
eggs	2	egg	
egg whites	66	egg_whites	66
fish	41	fish	66
tuna	1	fish	
salmon	24	fish	
avocado	8	fruit_fatty	40
olives	32	fruit_fatty	
banana	4	fruit_other	4
apple	45	fruit_tree	120
pears	22	fruit_tree	
grapes	51	fruit_tree	
figs	2	fruit_tree	
pineapple	65	fruit_tropical	84
mandarin oranges	11	fruit_tropical	
orange	5	fruit_tropical	
grapefruit juice	2	fruit_tropical	
lime	1	fruit_tropical	
wheat berry	27	grain_other	61
quinoa	14	grain_other	
millet	14	grain_other	
bulgur	4	grain_other	
oatmeal	1	grain_other	
couscous	1	grain_other	
celery	2	herb	24
parsley	8	herb	
cilantro	9	herb	
basil	2	herb	
thyme	3	herb	
spinach (cooked)	8	leafy_greens	327

Continued on next page

Original Label	Instance Count	New Label	Instance Count
spinach (raw)	91	leafy_greens	
arugula	89	leafy_greens	
mixed greens	67	leafy_greens	
kale	20	leafy_greens	
chard	18	leafy_greens	
bok choy	12	leafy_greens	
mustard greens	11	leafy_greens	
lettuce	5	leafy_greens	
tatsoi	2	leafy_greens	
garden salad	4	leafy_greens	
green beans	34	legumes_green	38
snow peas	4	legumes_green	
chickpeas	15	legumes_other	20
lentils	1	legumes_other	
white beans	3	legumes_other	
black beans	1	legumes_other	
beef	32	meat_red	98
steak	20	meat_red	
pork	46	meat_red	
watermelon	41	melon	121
cantaloupe	51	melon	
honeydew melons	29	melon	
mushroom	8	mushrooms	8
almonds	71	nuts_seeds	97
walnuts	11	nuts_seeds	
chia seeds	14	nuts_seeds	
pumpkin seeds	1	nuts_seeds	
pasta	6	pasta	7
rice noodles	1	pasta	
bell peppers	17	peppers	31
pepper	10	peppers	
jalapenos	4	peppers	
cheese pizza	93	pizza	125
pepperoni pizza	19	pizza	
pizza	13	pizza	
chicken	70	poultry	123
chicken thighs	19	poultry	
grilled chicken	7	poultry	
chicken breast	17	poultry	
ground turkey	4	poultry	
turkey bacon	6	poultry	
white rice	33	rice	78

Continued on next page

Original Label	Instance Count	New Label	Instance Count
brown rice	32	rice	
wild rice	3	rice	
country rice	3	rice	
pilaf	2	rice	
fried rice	5	rice	
carrot	102	root_vegetable	105
turnip	1	root_vegetable	
celery root	1	root_vegetable	
jicama	1	root_vegetable	
caesar salad	55	salad_prepared	66
pasta salad	1	salad_prepared	
chicken salad	3	salad_prepared	
tuna salad	7	salad_prepared	
sausage	31	sausage	46
chicken apple sausage	15	sausage	
chayote squash	1	squash_eggplant	67
squash	33	squash_eggplant	
zucchini	19	squash_eggplant	
eggplant	14	squash_eggplant	
cherry tomatoes	79	tomato	93
tomatoes	11	tomato	
sun dried tomatoes	3	tomato	
tofu	17	tofu	17
roasted potatoes	37	tuber	156
hash browns	6	tuber	
potatoes	17	tuber	
yam	30	tuber	
sweet potato	66	tuber	
asparagus	8	vegetable_other	16
radishes	6	vegetable_other	
kimchi	2	vegetable_other	
yogurt	3	yogurt	5
frozen yogurt	2	yogurt	

1.2 Implementation Configuration

Supplementary Table S2 lists the complete architectural, optimisation, and preprocessing configuration used across all experimental conditions, to support reproduction of the reported results.

Table S2. Complete implementation configuration used for all experimental conditions. A single configuration was used across the unsegmented, binary segmentation, and ingredient-level conditions; the only experimental variable was the input representation and the associated preprocessing variant.

Setting	Value
Architecture	
Backbone	ResNet-101, ImageNet pretrained (IMAGENET1K_V1)
Backbone output	`layer4`, 2048 channels
Backbone fine-tuning	All layers trainable (no freezing)
Channel attention	Squeeze-and-Excitation, reduction ratio $r = 16$
SE bottleneck	$2048 \rightarrow 128 \rightarrow 2048$
Spatial pooling	Adaptive average pooling to 1×1
FC1	$2048 \rightarrow 1024$, ReLU, dropout 0.2
FC2	$1024 \rightarrow 256$, ReLU, dropout 0.2
FC3	$256 \rightarrow 5$ (single shared output layer)
Outputs	Mass, calories, fat, carbohydrate, protein
Optimisation	
Optimiser	Adam (default β_1, β_2)
Initial learning rate	1×10^{-4}
Learning-rate schedule	ExponentialLR, $\gamma = 0.99$ per epoch
Weight decay	0
Gradient clipping	Global norm, maximum 1.0
Loss	Summed per-nutrient normalised MAE, $\epsilon = 1 \times 10^{-8}$
Epochs	300
Batch size	8
Checkpoint selection	Lowest mean PMAE on the evaluation split
Data and preprocessing	
Input resolution	384×384
Training augmentation	Random horizontal flip ($p = 0.5$); random resized crop to 384, scale $(0.8, 1.0)$, aspect ratio $(0.9, 1.1)$
Evaluation preprocessing	Resize shorter side to 384, centre crop 384×384
Normalisation	ImageNet channel mean and standard deviation
Background definition	All three channels $\leq 1/255$
Mean background fill	ImageNet channel mean, applied before normalisation
Bounding-box padding	1% of bounding-box size on each side
Environment and reproducibility	
Random seeds	0 (PyTorch, CUDA, NumPy, Python `random`)
cuDNN deterministic mode	Not enabled
Framework	Python 3.7, PyTorch 1.13
Hardware	Single NVIDIA Tesla T4 GPU

1.3 Train/Evaluation Ingredient Frequency

Supplementary Figure S1 shows the ingredient mask frequency for both the training and evaluation sets.

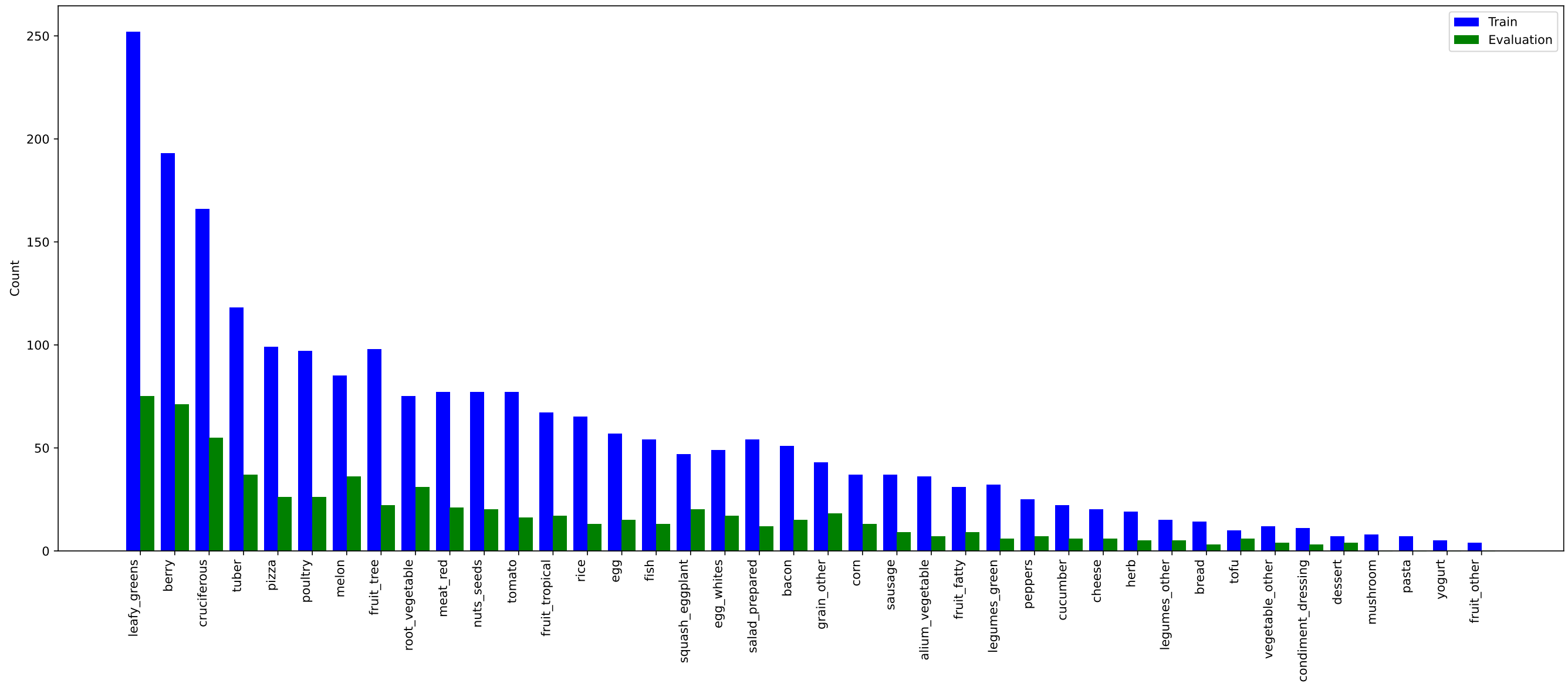

Figure S1: Ingredient (mask) frequency on the training and evaluation sets.

1.4 ImageNet Mean Background Replacement

Supplementary Figure S2 shows the results of the ImageNet-mean background replacement preprocessing condition.

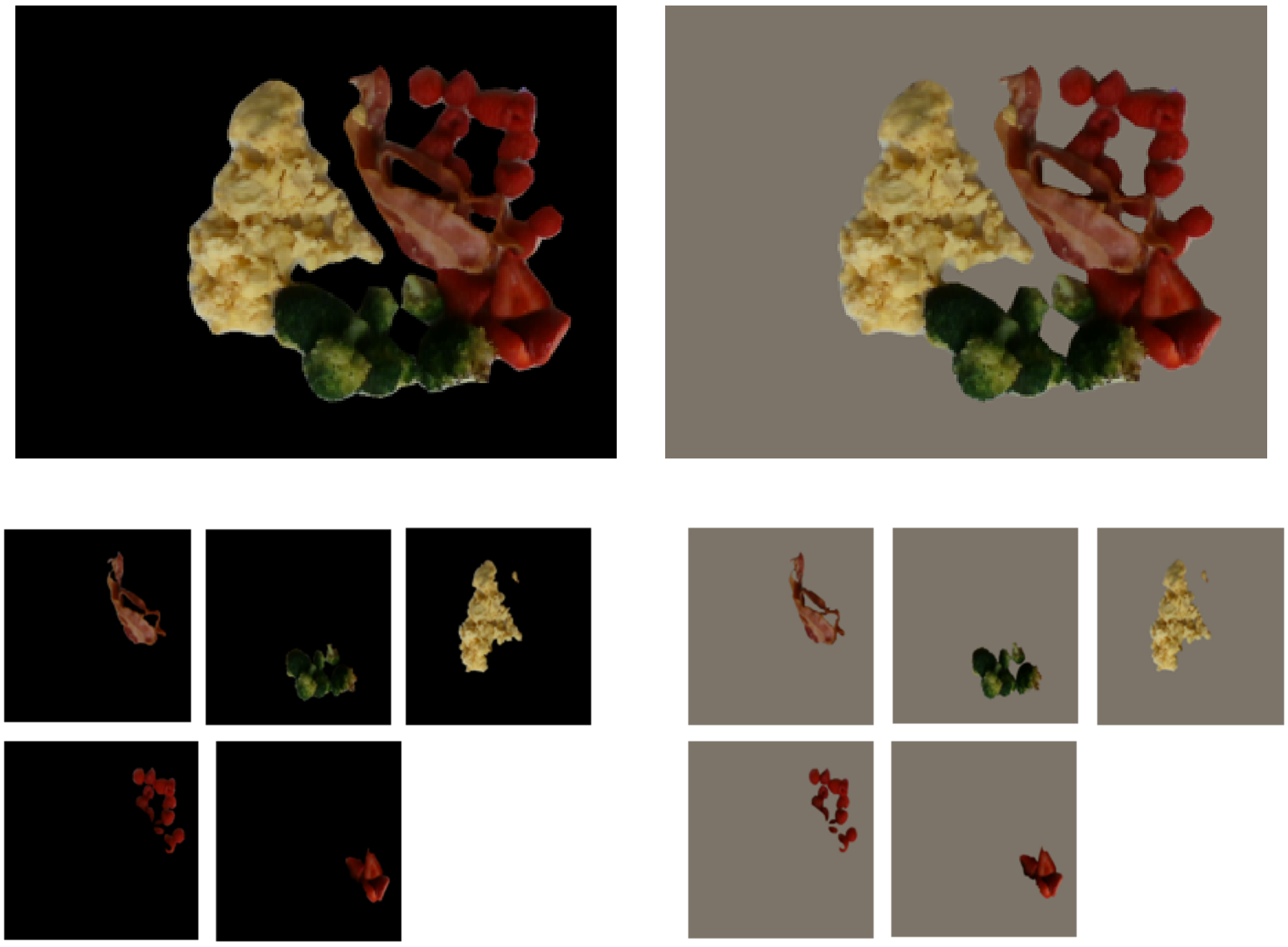

Figure S2: Effect of ImageNet mean background replacement. **(A)** Top: a binary-segmented dish before (left) and after (right) the ImageNet mean background fill is applied. **(B)** Bottom: ingredient instances from a dish before (left) and after (right) the fill is applied.

1.5 Foreground Bounding-Box Cropping

Supplementary Figure S3 shows the results of the foreground bounding-box cropping preprocessing condition.

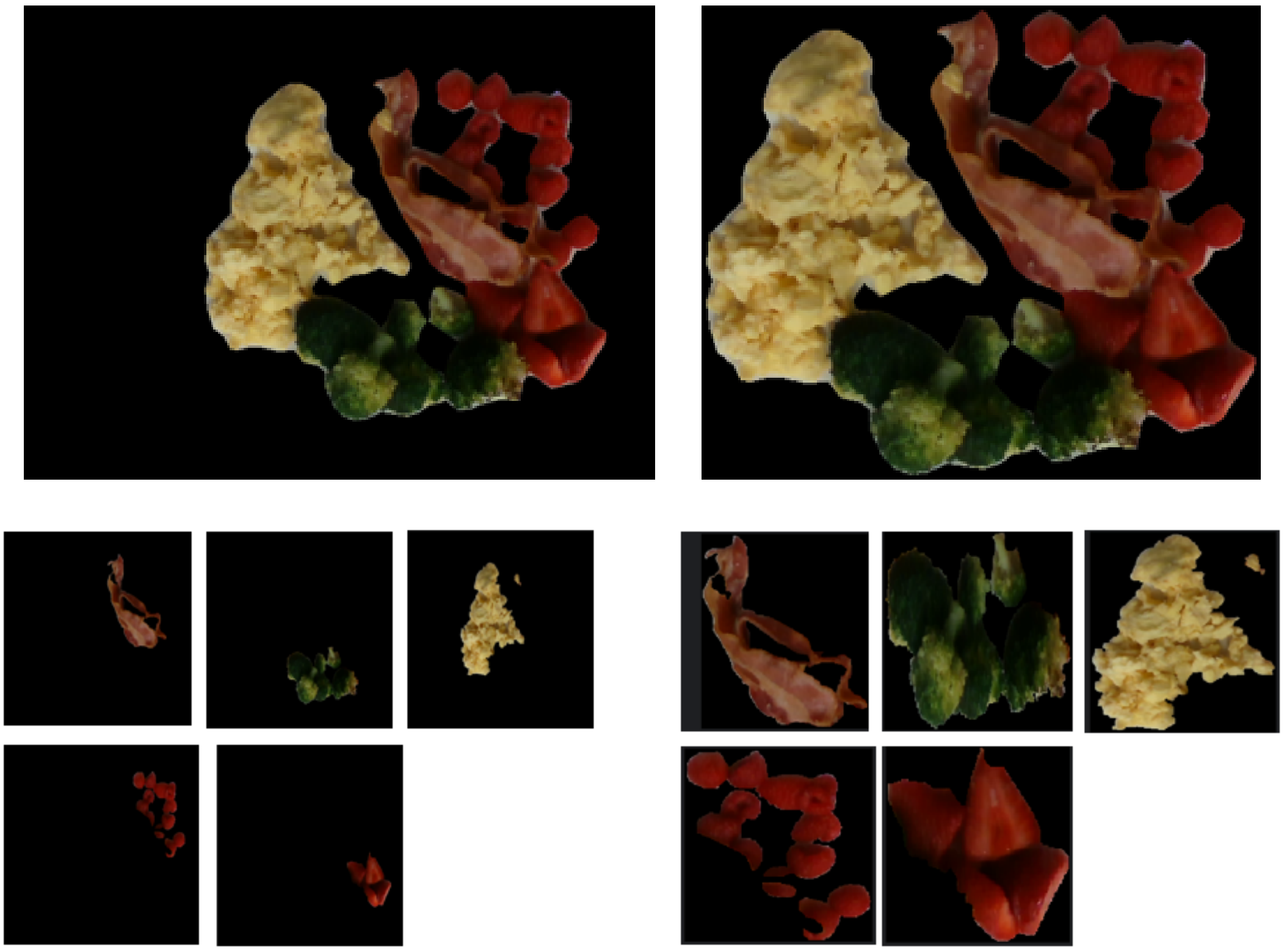

Figure S3: Effect of foreground bounding-box cropping. **(A)** Top: a binary-segmented dish before (left) and after (right) background removal via bounding-box generation and resizing. **(B)** Bottom: ingredient instances before (left) and after (right) the same process.

1.6 Cross-Validation of the Main Comparison

To assess whether the differences between segmentation conditions are systematic rather than an artifact of a single train-evaluation split, five-fold cross-validation for the three conditions under the standard preprocessing setting was performed. All 1,056 dishes were partitioned into five folds; folds were defined at the level of whole dishes, so that all ingredient segments of a given dish remained within a single fold, preventing information leakage in the ingredient-level condition. For each fold, a model was trained from scratch on the remaining four folds and evaluated on the held-out fold, using the same architecture, optimiser, and training schedule as the main experiments. Cross-validation was applied to the standard preprocessing condition only, which underlies the paper's headline comparison; the preprocessing ablation (Section 3.9.1 of main manuscript) experiments are still reported on the single split.

Table S3 reports the mean and standard deviation of PMAE across folds. The ranking observed in the main analysis was preserved in every fold: unsegmented input achieved the lowest error ($20.80 \pm 0.35\%$), binary segmentation was comparable but slightly higher ($21.96 \pm 0.84\%$), and ingredient-level segmentation was consistently the highest ($25.53 \pm 1.90\%$). Critically, the between-condition differences were large relative to the fold-to-fold variation: the gap between ingredient-level and unsegmented performance (4.73) exceeded the standard deviation of either condition across folds by a wide margin, and ingredient-level segmentation produced higher error than both other conditions in every fold. This indicates that the degradation associated with ingredient-level decomposition is systematic rather than a consequence of a particular data split. The cross-validated means are modestly higher than the single-split values reported in the main text, consistent

with the observation (Section 4.1 of the main manuscript) that the single-split figures, whose checkpoints were selected on the evaluation set, are optimistic estimates.

Table S3. Five-fold cross-validated mean PMAE (%) for the three segmentation conditions under standard preprocessing. Folds were defined over whole dishes (all 1,056 dishes), so that all ingredient segments of a dish remained within a single fold, preventing leakage in the ingredient-level condition. Values are mean PMAE $\pm$ standard deviation across the five folds. The ranking observed in the main analysis is preserved in every fold, and the between-condition differences are large relative to the fold-to-fold variation.

**Condition**	**Mean PMAE (%)**	**Per-fold PMAE (%)**
Unsegmented	20.80 ± 0.35	20.87, 20.36, 20.54, 21.21, 21.00
Binary segmentation	21.96 ± 0.84	20.96, 23.04, 21.83, 22.54, 21.42
Ingredient-level	25.53 ± 1.90	28.46, 26.27, 24.59, 24.71, 23.61

1.7 Per-Nutrient Ingredient Error Contributions

The top 10 ingredient groups contributing to mass, calorie, fat, carbohydrate, and protein prediction error are shown in Supplementary Figure S4.

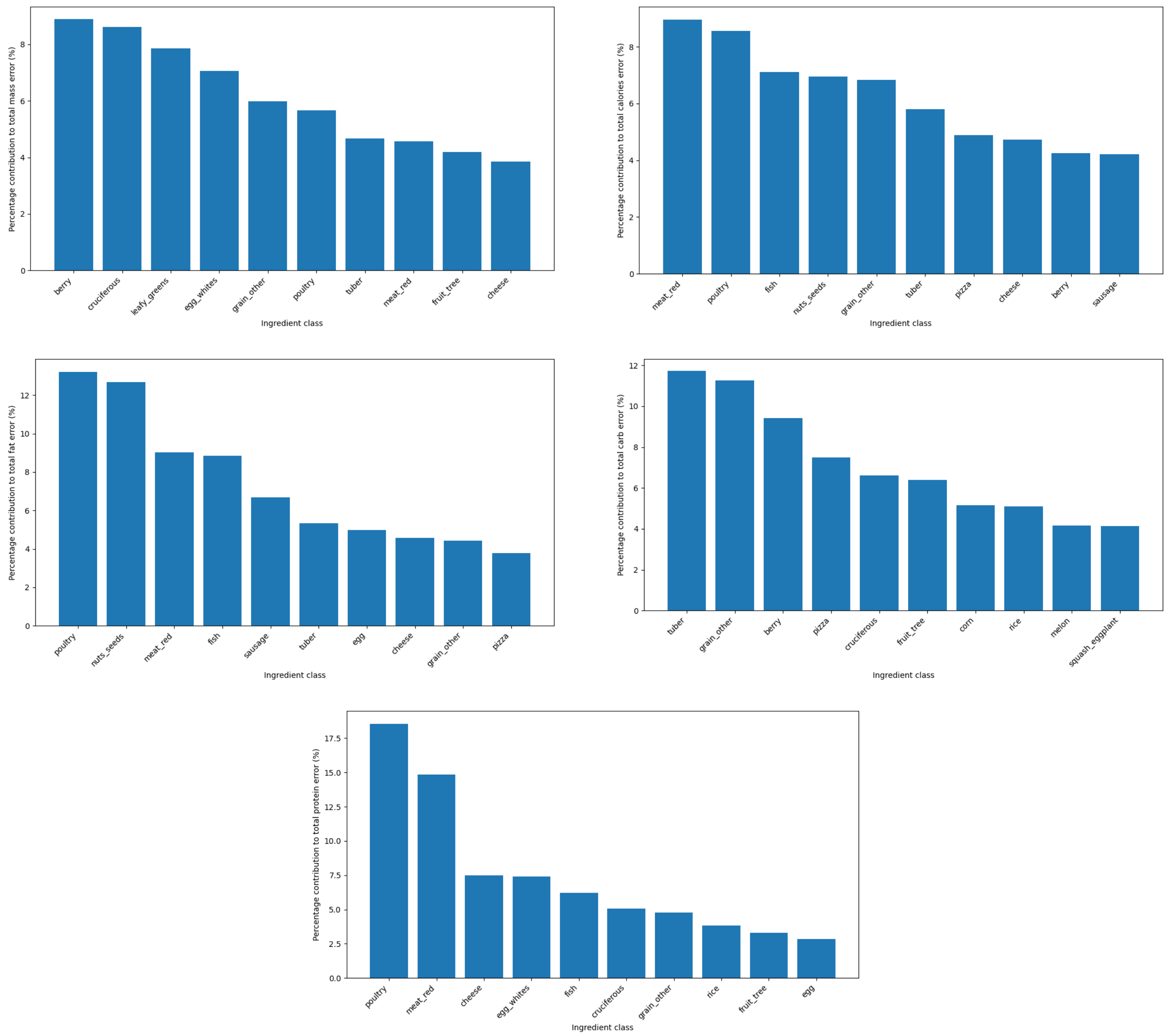

Figure S4: Top 10 ingredient groups contributing to prediction error for each nutrient. **(A)** Mass (top-left), **(B)** calories (top-right), **(C)** fat (middle-left), **(D)** carbohydrate (middle-right), and **(E)** protein (bottom).

1.8 FoodSAM segmentation performance

FoodSAM was trained and evaluated on the dataset using the same split described in the main manuscript. Supplementary Table S4 reports per-class segmentation performance (IoU and accuracy), and Supplementary Table S5 summarizes the overall performance (mIoU, mAcc, aAcc) across the evaluation set. The classes fruit_other, mushroom, pasta, and yogurt were excluded from evaluation due to having fewer than 10 instances.

Table S4. Per-class segmentation performance of FoodSAM evaluated on the extended Nutrition5K ingredient-level dataset.

Class	IoU	Acc
background	98.74	99.38
alium_vegetable	0.00	0.00
bacon	72.65	75.99
berry	80.83	87.46
bread	55.27	57.53
cheese	40.78	42.27
condiment_dressing	78.67	81.37
corn	73.84	78.28
cruciferous	75.12	89.74
cucumber	11.31	21.01
dessert	39.70	40.34
egg	80.98	90.97
egg_whites	92.42	95.22
fish	39.55	53.59
fruit_fatty	58.41	61.89
fruit_tree	86.95	95.32
fruit_tropical	70.84	86.96
grain_other	17.55	29.17
herb	0.00	0.00
leafy_greens	80.82	92.38
legumes_green	80.35	88.02
legumes_other	8.65	11.01
meat_red	51.23	72.54
melon	77.39	85.49
nuts_seeds	86.76	93.20
peppers	0.00	0.00
pizza	84.96	93.59
poultry	56.79	72.79
rice	33.18	58.84
root_vegetables	59.84	65.25
salad_prepared	85.27	97.2
sausage	61.35	76.99
squash_eggplant	61.49	70.06
tofu	8.74	9.90
tomato	61.75	65.07
tuber	70.64	92.06
vegetable_other	47.58	54.77

Table S5. Overall segmentation performance of FoodSAM evaluated the extended Nutrition5K ingredient-level dataset.

Scope	mIoU	mAcc	aAcc
Global	55.01	64.48	95.74

1.9 Full-Dataset Comparison of Unsegmented and Binary Segmentation

Supplementary Table S6 reports the mean and per-nutrient PMAE obtained when the unsegmented and binary segmentation conditions were trained and evaluated on the full 3,224-image overhead RGB set, using the standard preprocessing condition.

Table S6. Mean and per-nutrient PMAE (%) for the unsegmented and binary segmentation conditions trained and evaluated on the full 3,224-image overhead RGB set, under standard preprocessing. The ingredient-level condition is not included, as ingredient-level annotations are available only for the 1,056-image subset. Lower values indicate better performance; the best value in each column is shown in bold.

Condition	Mean	Mass	Calories	Fat	Carbohydrate	Protein
Unsegmented	**14.99**	**9.75**	**13.97**	**19.16**	**15.90**	**16.16**
Binary Segmentation	17.19	11.70	15.92	21.55	18.59	18.16

Note. Both conditions used the same architecture, optimiser, and training schedule described in Section 3.5 of the main manuscript, with identical treatment of the two conditions within this experiment. As in the main experiments, the reported checkpoint was selected on the evaluation split (see Section 3.7 of main manuscript); absolute values are therefore not directly comparable to the results reported in Table 1 of the main manuscript, which used a smaller training set.

1.10 Feature-Space Separability of Grouped Labels

A potential concern regarding the ingredient-level results is that consolidating the 145 original ingredient labels into 40 broader groups may itself have increased the difficulty of ingredient-level regression by assigning visually heterogeneous sub-classes (for example, the five berry types) to a common target. To assess this, we quantified the separability of the original sub-classes within each merged group in the trained model's learned feature space.

For each ingredient instance, the 2,048-dimensional feature vector immediately preceding the regression head of the trained ingredient-level model was extracted. Sub-class separability within each merged group was then quantified using the silhouette coefficient, computed from these feature vectors using Euclidean distance. For an instance i, let $a(i)$ denote the mean Euclidean distance between i and all other instances of the same original sub-class, and let $b(i)$ denote the minimum mean distance between i and the instances of any other original sub-class within the same merged group. The silhouette coefficient for instance i is:

$$s(i) = \frac{b(i) - a(i)}{\max\{a(i),\, b(i)\}}, \tag{S1}$$

where $s(i)$ ranges from -1 to 1. Values near zero or below indicate substantial overlap between sub-classes, whereas values approaching 1 indicate greater separation. The mean silhouette coefficient was computed for each of the 34 merged groups containing more than one original label. Separability was assessed directly in the 2,048-dimensional feature space; two-dimensional t-SNE projections of representative groups were generated separately, for visualisation only.

Across the 34 multi-class groups, the median silhouette coefficient was -0.003 (mean -0.017), indicating little to no separation between constituent sub-classes. The lowest coefficients were observed for several of the most frequent groups, including *leafy_greens* (-0.400), *alium_vegetable* (-0.389), *rice* (-0.360), and *berry* (-0.199), indicating substantial overlap in feature space. The few groups with positive coefficients were predominantly low-frequency groups, for which silhouette estimates are less reliable and for which data sparsity motivated the grouping procedure in the first place. Silhouette coefficients for all merged groups are reported in Table S7, and representative t-SNE projections are shown in Figure S5.

Together, these results indicate that label grouping did not force visually distinct inputs onto shared targets, and is therefore unlikely to be the primary driver of the higher ingredient-level error. This is consistent with the preservation of macronutrient composition at the ingredient level (main manuscript Section 4.7) and with the observed increase in prediction error with ingredient count (main manuscript Section 4.6).

Table S7. Sub-class separability within the 34 merged ingredient groups containing multiple original labels, measured by the mean silhouette coefficient of the 2,048-dimensional penultimate-layer features of the trained ingredient-level model (Euclidean distance). n denotes the number of ingredient instances and k the number of original sub-classes. Groups are ordered from lowest to highest silhouette coefficient.

**Group**	n	k	**Silhouette**
leafy_greens	321	11	−0.400
alium_vegetable	42	4	−0.389
rice	75	6	−0.360
pasta	6	2	−0.328
legumes_other	20	4	−0.271
root_vegetable	81	3	−0.207
berry	258	5	−0.199
fruit_tropical	84	5	−0.164
pizza	124	3	−0.144
herb	23	5	−0.141
poultry	121	6	−0.132
squash_eggplant	64	4	−0.090
cheese	26	6	−0.079
cruciferous	207	4	−0.057
grain_other	56	6	−0.037
tuber	155	5	−0.033
meat_red	97	3	−0.009
tomato	83	3	0.004
melon	120	3	0.004
fish	65	3	0.014
peppers	30	3	0.016
fruit_tree	117	4	0.033
fruit_fatty	40	2	0.053
egg	70	2	0.064
condiment_dressing	13	5	0.089
sausage	46	2	0.108
nuts_seeds	93	4	0.161
corn	50	4	0.200
dessert	11	3	0.233
salad_prepared	62	4	0.237
legumes_green	35	2	0.260
bread	17	7	0.274
vegetable_other	16	3	0.325
yogurt	5	2	0.394
**Median**			−0.003
**Mean**			−0.017

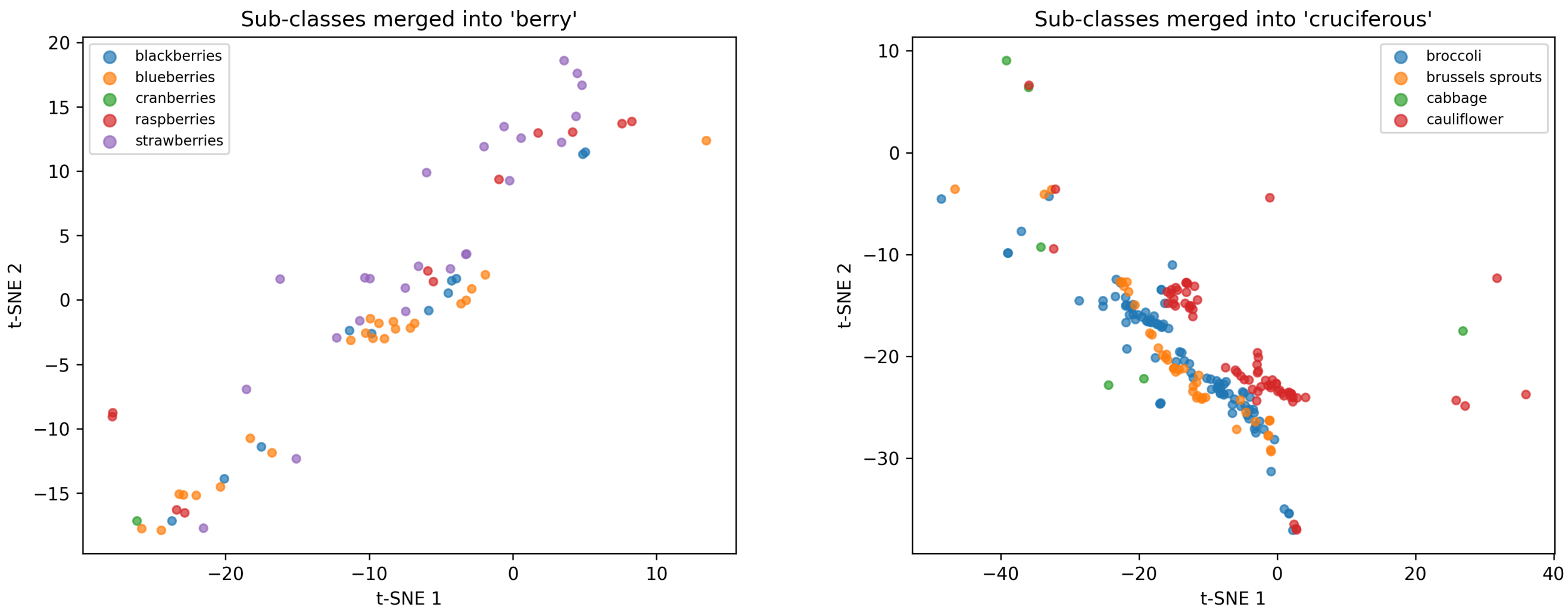

Figure S5: Two-dimensional t-SNE projections of the penultimate layer features for two representative merged groups: **(left)** the *berry* group (five sub-classes) and **(right)** the *cruciferous* group (four sub-classes). In both, the original sub-classes are broadly intermixed rather than forming separate clusters, consistent with the low silhouette coefficients reported in Table S7. Projections are shown for visualisation only; separability was quantified in the full 2,048 dimensional feature space.

1.11 Per-Sub-Ingredient Error Within Merged Classes

To examine more directly whether consolidating the 145 original ingredient labels into 40 merged classes affected regression performance, the ingredient-level model's error was broken down by original sub-ingredient within five of the largest merged classes. For each ingredient instance, the model's prediction was matched to its original (pre-merge) sub-ingredient label, and the z-normalised mean absolute error (using the ingredient-level training statistics) was computed per sub-ingredient. The analysis was performed over the evaluation-set predictions. Sub-ingredients with at least eight evaluation-set instances are shown individually; those with fewer are pooled into an "other" row, as per-sub-ingredient estimates from few instances may be statistically unreliable. Results are given in Table S8.

Among sub-ingredients with at least eight instances, the constituent sub-ingredients were predicted with broadly similar error, despite being visually distinct (for example, the five berry types). Some mild variation was observed, such as cauliflower, which showed a somewhat higher error than its cruciferous class-mates - however this is consistent with ingredient-specific differences in the difficulty of quantity estimation rather than with a systematic penalty introduced by grouping. Sub-ingredients with few instances, pooled as "other", showed more variable error, as expected from small samples, but are not reliably estimated individually. Importantly, no well-populated sub-ingredient exhibited the large error inflation that would be expected if merging had forced the model to reconcile visually distinct items that it could not represent consistently. These results complement the silhouette analysis of Section 1.10: the sub-classes are neither separably represented in the model's feature space nor predicted with markedly different accuracy, indicating that the 145-to-40 grouping is unlikely to be a primary driver of the ingredient-level model's higher error.

Table S8. Ingredient-instance-level z-normalised mean absolute error (MAE) of the ingredient-level model, broken down by original sub-ingredient within five of the largest merged classes. Errors are normalised per nutrient by the ingredient-level training standard deviation. n is the number of ingredient instances; rows in italics give the pooled value for the whole merged class. Only sub-ingredients with at least eight instances are shown individually. Sub-ingredients with less than eight instances are contained within the ”other” class.

Merged class	Sub-ingredient	n	Mass	Cal.	Fat	Carb	Prot.	Mean
berry	strawberries	24	0.27	0.07	0.02	0.18	0.02	0.11
	blueberries	23	0.16	0.07	0.03	0.15	0.01	0.08
	blackberries	11	0.19	0.05	0.01	0.16	0.01	0.08
	raspberries	11	0.22	0.07	0.01	0.22	0.01	0.11
	other ($n < 8$, 1 sub-ingredients)	1	0.02	0.01	0.00	0.03	0.00	0.01
	**all berry**	70	0.21	0.07	0.02	0.17	0.01	0.10
cruciferous	broccoli	26	0.27	0.06	0.01	0.15	0.04	0.11
	cauliflower	14	0.30	0.12	0.05	0.20	0.19	0.17
	brussels sprouts	10	0.28	0.06	0.01	0.16	0.06	0.11
	other ($n < 8$, 1 sub-ingredients)	1	0.14	0.33	0.17	0.06	0.56	0.25
	**all cruciferous**	51	0.28	0.08	0.02	0.17	0.10	0.13
tuber	sweet potato	15	0.21	0.08	0.09	0.30	0.03	0.14
	roasted potatoes	10	0.12	0.12	0.15	0.14	0.03	0.11
	other ($n < 8$, 2 sub-ingredients)	12	0.28	0.32	0.10	0.75	0.05	0.30
	**all tuber**	37	0.21	0.17	0.11	0.40	0.04	0.19
melon	cantaloupe	14	0.19	0.05	0.00	0.17	0.01	0.09
	watermelon	12	0.14	0.05	0.00	0.15	0.01	0.07
	honeydew melons	9	0.15	0.04	0.00	0.13	0.01	0.07
	**all melon**	35	0.16	0.05	0.00	0.15	0.01	0.07
leafy_greens	arugula	24	0.18	0.03	0.01	0.05	0.03	0.06
	spinach (raw)	17	0.20	0.03	0.01	0.05	0.03	0.06
	mixed greens	14	0.18	0.03	0.01	0.07	0.03	0.06
	other ($n < 8$, 7 sub-ingredients)	17	0.17	0.04	0.01	0.07	0.02	0.06
	**all leafy_greens**	72	0.18	0.03	0.01	0.06	0.03	0.06
